# Self-decoupled radiofrequency coils for magnetic resonance imaging

**DOI:** 10.1038/s41467-018-05585-8

**Published:** 2018-08-28

**Authors:** Xinqiang Yan, John C. Gore, William A. Grissom

**Affiliations:** 1 Vanderbilt University Institute of Imaging Science, Nashville, TN 37232 USA; 2 Department of Radiology and Radiological Sciences, Vanderbilt University, Nashville, TN 37232 USA; 3 Department of Biomedical Engineering, Vanderbilt University, Nashville, TN 37232 USA; 4 Department of Electrical Engineering, Vanderbilt University, Nashville, TN USA

## Abstract

Arrays of radiofrequency coils are widely used in magnetic resonance imaging to achieve high signal-to-noise ratios and flexible volume coverage, to accelerate scans using parallel reception, and to mitigate field non-uniformity using parallel transmission. However, conventional coil arrays require complex decoupling technologies to reduce electromagnetic coupling between coil elements, which would otherwise amplify noise and limit transmitted power. Here we report a novel self-decoupled RF coil design with a simple structure that requires only an intentional redistribution of electrical impedances around the length of the coil loop. We show that self-decoupled coils achieve high inter-coil isolation between adjacent and non-adjacent elements of loop arrays and mixed arrays of loops and dipoles. Self-decoupled coils are also robust to coil separation, making them attractive for size-adjustable and flexible coil arrays.

## Abstract (web summary)

Conventional coil arrays require complex decoupling technologies to reduce electromagnetic coupling between coil elements. Here, the authors report a self-decoupled RF coil design that achieves high inter-coil isolation between adjacent and non-adjacent elements and mixed arrays of loops and dipoles

## Introduction

Magnetic resonance imaging (MRI) is a widely used non-invasive medical imaging modality that provides a variety of high-resolution images of the human body, with versatile soft-tissue and functional contrast^1^. The radiofrequency (RF) coils used in an MRI scanner to transmit RF energy into the body and receive signals from it play a critical role in determining image quality in terms of signal-to-noise ratio (SNR) and image uniformity, as well as other technical constraints on scanner performance. For three decades, arrays of receive coils have been used in MRI to achieve flexible coverage of large imaging volumes with high SNR^2,3^, and to accelerate scans using parallel imaging techniques^4–8^. Advances in RF array performance have usually come through increasing the number of coils in the array^6,9,10^. For example, receive arrays with large numbers of coils (≥32) have been fundamental to recent advances in the speed, sensitivity and resolution of neuroimaging^8,11,12^. 3 Tesla MRI scanners used for clinical applications are today equipped with 32 receiver channels as standard, and array coils are used exclusively for signal reception. Array coils are also used for RF transmission at ultra-high magnetic field strengths (7 Tesla and higher), as a means to achieve spatially homogeneous transmit RF fields in a patient-adaptive manner, while controlling tissue heating (quantified as global and local specific absorption rate (SAR)^13–17^). In that application, increasing the number of coils in the array improves power efficiency and image uniformity. Current 7 Tesla scanners are typically equipped with 8 transmit channels, and there have been reports of systems developed with as many as 32^18^ channels. Even for scanners with a smaller number of transmit channels, the benefits of many-coil transmit arrays can be realized using array-compression networks^19^.

The most widely used RF coil array element is a resonant loop coil comprising a circle or rectangle of copper wire or tape, which is uniformly segmented along its length by identical impedances (usually capacitors) to achieve a uniform current distribution and avoid antenna effects. When assembled into an array, these loops electromagnetically couple to each other due to magnetic flux linkage, which is referred to as loop-mode coupling. Minimizing this coupling is a central challenge in building RF coil arrays with many coil elements. When receiving RF signals from the body, strong coupling can lead to noise amplification and may limit the degree to which image acquisition can be accelerated using parallel imaging. When transmitting RF energy into the body, coupled power is absorbed by protection circuits and wasted. These and other power losses necessitate the use of very large RF amplifiers, with high cost and siting requirements. Transmit coil coupling also limits the ability to control the shape of the RF fields produced within the subject being scanned, because each coil’s transmit field profile becomes less distinct from those of its neighbours.

The most common strategy to minimize array coil coupling is to partially overlap adjacent loops. In receive coil arrays, overlapping is combined with low-impedance preamplifier decoupling, which minimizes coupling between non-adjacent elements^3^. Other less common decoupling methods have been described including transformers^20^, inter-connecting capacitive/inductive networks^21–26^, and passive resonators^27–30^. However, these methods increase the complexity of dense coil arrays by adding circuitry or restricting array geometry, and most cannot simultaneously decouple adjacent and non-adjacent elements. They are also incompatible with recently proposed RF arrays comprising mixtures of loops, dipoles, and monopoles, which have the potential to increase receive sensitivity and transmit performance by providing access to more diverse field modes^10,31–33^. Transmit arrays can be decoupled by driving each element with special RF amplifiers configured as current sources^34,35^ or using Cartesian feedback circuits^36,37^, but these approaches require new amplifier designs and can limit power efficiency, linearity and bandwidth, so they have not yet been adopted in commercial scanners. The need for robust flexible body and extremity arrays has recently led to the development of coils based on novel materials and new approaches to preamplifier decoupling^38,39^. These coils have dramatically improved decoupling and loading robustness compared to traditional coils, but they also require specialized manufacturing and have been demonstrated only in receive arrays due to their dependence on preamplifier decoupling.

**Fig. 1:**
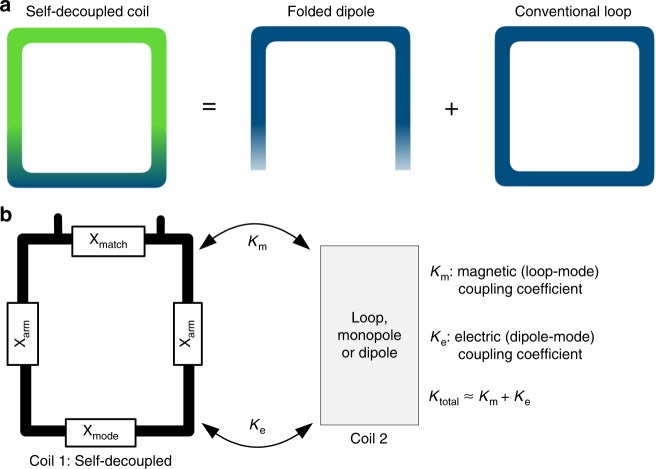
Self-decoupled coil current distributions and coupling. **a** A self-decoupled coil with a relatively large impedance in the bottom conductor has a non-uniform current distribution, which is equivalent to a superposition of conventional loop and folded dipole currents. **b** Proximal loop coils couple magnetically to each other, and proximal dipole antennas couple electrically to each other. When a self-decoupled loop coil (Coil 1) is placed next to another coil (Coil 2), they couple both magnetically and electrically. If the magnetic and electric coupling coefficients *K*_m_ and *K*_e_ can be tuned to have opposite signs and equal magnitudes by adjusting the X_mode_ impedance in Coil 1, the total coupling is zero, and Coil 1 is self-decoupled from Coil 2

Here we propose a simple and practical RF coil design for arrays called the “self-decoupled” coil, which retains the loop structure of conventional coils and does not constrain array geometry or require additional circuitry. In this design, instead of uniformly segmenting the coil’s conductor with capacitors, a relatively large impedance (X_mode_) is positioned opposite the coil’s feed port so that the coil behaves as a combination of a loop and a folded dipole superimposed on each other^40^, as illustrated in Fig. 1a. Proximal loop coils couple magnetically to each other, and proximal dipole antennas couple electrically to each other. Thus, coupling between a self-decoupled coil and a second coil is a mixture of magnetic (loop-mode) and electric (dipole-mode) coupling, as illustrated in Fig. 1b. If the magnetic (*K*_m_) and electric (*K*_e_) coupling coefficients have the same magnitude and opposite signs, they will cancel and the coil will be self-decoupled from other coils. The self-decoupled concept can be used to decouple adjacent or non-adjacent elements, and applies to both transmit and receive arrays. It can also be used for arrays of mixed types such as loop plus dipole arrays. The unique structure of a self-decoupled coil makes its decoupling performance much more robust as a function of the distance between coil elements compared to conventional coils. With this feature, the self-decoupled design can improve the performance of size-adjustable and flexible coils used to match individual patients and improve patient comfort.

## Results

### Balancing magnetic and electric coupling

**Fig. 2:**
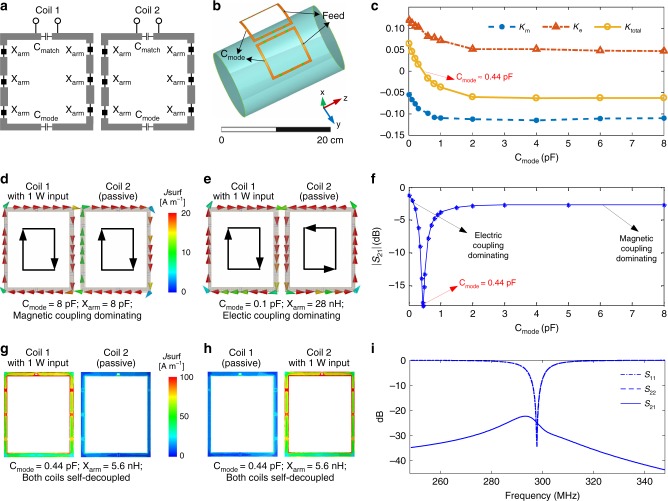
Self-decoupling a two-loop array. **a** Schematic of a pair of self-decoupled coils. **b** Electromagnetic simulation model of a pair of self-decoupled coils. The magnetic and electric coupling are balanced by tuning the C_mode_ capacitors, while adjusting the X_arm_ impedances to maintain coil tuning, and the C_match_ capacitors to maintain a coil input impedance of 50 Ohms. **c** Calculated magnetic and electric coupling coefficients (*K*_m_ and *K*_e_) versus C_mode_. **d** Simulated vector current distributions of magnetic coupling-dominated coils. **e** Simulated vector current distributions of electric coupling-dominated coils. In both cases, Coil 1 is excited with 1 Watt and its current flows clockwise along its conductors. **f** The coils’ transmission coefficient (|*S*_21_|) versus C_mode_. Coupling is minimized and the coils are self-decoupled for C_mode_ = 0.44 pF. **g** Simulated current distributions of the self-decoupled coils with Coil 1 excited. **h** Simulated current distributions of the self-decoupled coils with Coil 2 excited. In both cases, the residual currents due to coupling in the passive coils are negligible. **i** Simulated scattering parameters versus frequency for the self-decoupled coils

We simulated and built self-decoupled coils with a capacitor C_mode_ that was positioned opposite to the feed port (illustrated in Fig. 2a), to adjust the impedance of the bottom coil conductor and balance the magnetic and electric coupling. When changing C_mode_, the coils’ resonance frequencies (298 MHz for 7 Tesla MRI) and input impedance were maintained by tuning the impedances X_arm_ (which were capacitors or inductors) and the capacitor C_match_. Figure 2b shows an electromagnetic simulation model of a pair of 10 × 10 cm^2^ loops separated by 1 cm. Figure 2c plots the magnetic coupling coefficients (*K*_m_) and the electric coupling coefficients (*K*_e_) as a function of C_mode_, which were calculated using the methods of Hong et al.^41^ and Chu et al.^42^; further details are provided in Supplementary Table 1 and Equations 1-3. Note that the *K*_e_ definition used here represents electric coupling via free space, and unlike the definition used in Roemer et al.^3^, it is primarily a reactive coupling and does not include resistive coupling through the conductive sample. Figure 2c, d, e shows that setting C_mode_ to a large value such as 8 pF in both coils (corresponding to low impedance and thus a relatively large current in the bottom rung) causes the magnetic coupling to dominate, while setting C_mode_ to a small value such as 0.1 pF (corresponding to high impedance and thus a relatively small current in the bottom rung) causes the electric coupling to dominate. The currents in the two loops are of the same magnitude and opposite directions in magnetic- and electric-coupling-dominated cases, which validates that the two coupling coefficients have opposite signs. The negative sign of the magnetic coupling coefficients is consistent with Lenz’s law, which dictates that the current induced in the passive coil will generate magnetic flux to cancel out the active magnetic flux through it. As C_mode_ decreases, the two self-decoupled coils increasingly behave as a pair of co-linear folded dipoles that couple electrically, and the positive sign of the electric coupling coefficients is consistent with prior results on the mutual coupling between co-linear dipoles separated by less than 1/10 of a wavelength^43^. Figure 2f shows that the mutual coupling is minimized and the coils are self-decoupled when C_mode_ ~ 0.44 pF so that *K*_e_ = −*K*_m_. Figure 2g, h, i further shows current distributions and scattering (S−) parameter plots when the coils are self-decoupled.

### Constructed self-decoupled coils

Although the required C_mode_ can be obtained through a simulation analysis such as in Fig. 2, it can also be tuned on the benchtop based on *S*_21_ measurements. Specifically, to achieve the self-decoupled condition, C_mode_ should be adjusted so that the frequency with minimum *S*_21_ (denoted *f*_m_) is equal to the Larmor frequency (*f*_0_). In the Supplementary Information we show that $f_{\mathrm{m}} = \sqrt {\left| {K_{\mathrm{e}}} \right|/\left| {K_{\mathrm{m}}} \right|} f_0$, so when *f*_m_ < *f*_0_, magnetic coupling dominates and C_mode_ should be decreased, and when *f*_m_ > *f*_0_, electric coupling dominates and C_mode_ should be increased. This process is illustrated in Supplementary Figure 1 for two initial values of C_mode_. In an array with more than two elements, the optimal C_mode_ for a two-coil array can be used as an initial value for all coils. As each new self-decoupled coil is added to the array, the C_mode_ and X_arm_ of its neighbours (in practice, mainly X_arm_) may then be re-tuned.

**Fig. 3:**
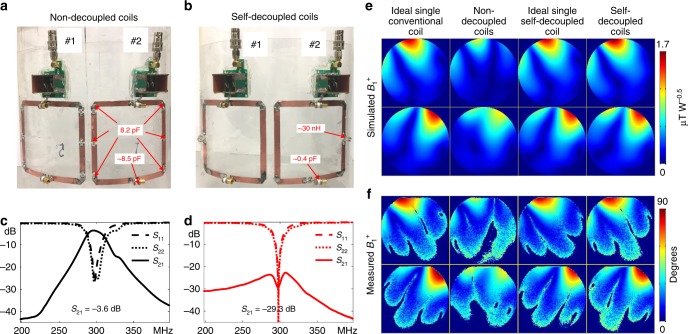
Two-loop coil array comparison. Constructed two-loop non-decoupled conventional (**a**) and self-decoupled (**b**) coil arrays are with the same dimensions as the simulated coils in Fig. 2. **c** Measured scattering (S-) parameter plots of the non-decoupled coils. **d** Measured S-parameter plots of the self-decoupled coils. **e**, **f** Simulated and measured axial RF transmit field strength (*B*_1_^+^) maps of ideal single coils, the two non-decoupled loops, the ideal single self-decoupled coils and the two self-decoupled coils. Supplementary Figure 4 shows an additional comparison to a pair of transformer-decoupled coils

A pair of constructed self-decoupled coils (loop–loop configuration) with the same sizes as the simulated coils are shown in Fig. 3a, b, along with a pair of conventional non-decoupled coils. Figure 3c, d plots the measured S-parameters of the two arrays. As expected, the non-decoupled coils are strongly coupled to each other, with a transmission coefficient *S*_21_ of −3.6 dB (i.e., power cross-talk of 44%). The strong coupling also makes the reflection coefficient (*S*_11_) plot asymmetrical and splits the resonance peaks. However, the coupling is only −29.3 dB (i.e., power cross-talk of 0.1%) between the two self-decoupled coils. Figure 3e, f shows that the RF transmit field strength (*B*_1_^+^) maps for each coil element match the ideal maps (where only one coil is present) for the self-decoupled coils, and that they have ~70 and ~47% greater power efficiency than the conventional non-decoupled coils. Note that the measured self-decoupled *S*_21_ is better than the simulated one in Fig. 2f, because the C_mode_ capacitors could be tuned continuously in the constructed coils but only discretely in the simulation. Simulated and measured multi-slice *B*_1_^+^ maps in multiple axial planes across the coil lengths are shown in Supplementary Figures 2 and 3, respectively. Although the current of the self-decoupled coils is non-uniform along the conductor length (stronger near the feed port and weaker near the C_mode_ capacitor), the slice-by-slice *B*_1_^+^ measurements show negligible decay along the z-direction. This can be understood by considering that *B*_1_^+^ = (*B*_x_ + i*B*_y_) / 2^44^ is mainly produced by the current on the vertical conductor segments arms for these square coils, where the current distribution is relatively uniform. The maximum 10-gram local SARs of the ideal single coils and the self-decoupled coils were 1.35 W Kg^-1^ and 1.66 W Kg^-1^, respectively. As described later, the SAR of the self-decoupled coil can be reduced to 1.44 W Kg^-1^ by using multiple larger C_mode_ capacitors placed in series. Overall, the self-decoupled coils were nearly identical to ideal conventional coils in isolation, in terms of RF transmit field and SAR efficiency.

A pair of transformer-decoupled (also referred to as inductively decoupled) coils^20^ was also built for comparison, as shown in Supplementary Figure 4. Like the self-decoupled coils, the transformer-decoupled coils had much lower (−19.1 dB) coupling than the non-decoupled coils and similar *B*_1_^+^ maps as the ideal single conventional coils. However, transformer decoupling is extremely sensitive to coil separation and is limited to loop arrays, while (as will be shown later) self-decoupling is insensitive to changes in coil separation and can be applied to non-adjacent elements and mixed dipole and loop arrays.

### Mixed element-type self-decoupled arrays

**Fig. 4:**
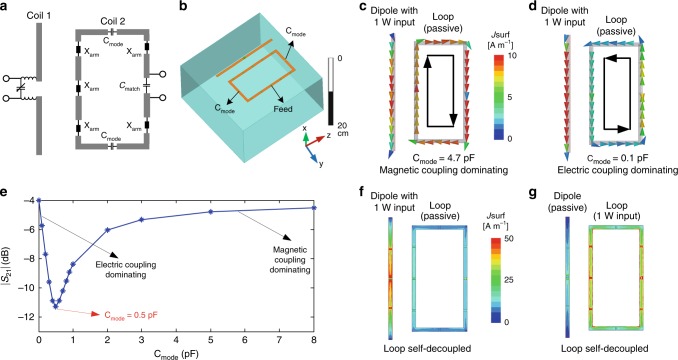
Self-decoupling a loop–dipole array. **a** Schematic of a dipole next to a self-decoupled loop coil. **b** Simulation model of a dipole next to a self-decoupled loop coil. **c**, **d** Simulated current distributions of loop-mode and dipole-mode coupling achieved by adjusting the two C_mode_ capacitors in the loop. **e** The coils’ transmission coefficient (|*S*_21_|) versus C_mode_. Coupling is high for both electric coupling-dominating (C_mode = _0.1 F) and magnetic coupling-dominating (C_mode_ = 4.7 pF) cases, but is minimized when the two kinds of coupling are balanced (C_mode_ = 0.5 pF). **f**, **g** Simulated current distributions when the magnetic coupling and electric coupling are balanced. The residual currents may be due to resistive coupling through the conductive phantom (*б* = 0.7 S m^−1^)

**Fig. 5:**
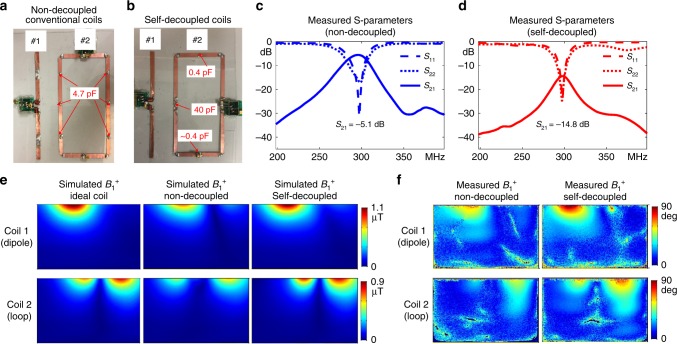
Constructed self-decoupled loop and dipole array. The constructed two-element conventional non-decoupled (**a**) and self-decoupled (**b**) coil arrays are with the same dimensions as the simulated coils in Fig. 4. **c** Measured scattering (S-) parameter plots of a two-element array comprising a dipole plus a conventional loop coil. **d** Measured S-parameter plots of a two-element array comprising a dipole plus a self-decoupled loop coil. Coupling between the coils was reduced from −5.1 dB to −14.8 dB (power cross-talk reduced from 31 to 3.3%) in the self-decoupled design. **e**, **f** Simulated and measured axial RF transmit field strength (*B*_1_^+^) maps of the dipole and loop without and with the self-decoupled design

The self-decoupled design was applied to a loop in a two-element loop–dipole array, shown in Fig. 4a, b. Although a loop coil is intrinsically decoupled from a radiative antenna such as a dipole or monopole when that antenna is placed across the loop’s center, in an array there is strong coupling between loops and adjacent dipoles/monopoles, especially when the loop coils are closely spaced or overlapped^31,32^. Figure 4c, d shows that, as in the loop–loop case, the currents induced in the loop coil have opposite directions for C_mode_ capacitors of very large versus very small values, which means the coupling coefficients *K*_m_ and *K*_e_ have opposite signs. Thus, C_mode_ can again be tuned to balance the two kinds of coupling and minimize total coupling (Fig. 4e, f, g). The experimental results in Fig. 5c, d show that the isolation between the loop and dipole was improved from −5.1 to −14.8 dB using the self-decoupled design. Compared to the non-decoupled case, the dipole and self-decoupled loop also have ~39% and ~24% greater power efficiency, respectively (Fig. 5e, f).

### Coil robustness to separation and loading

**Fig. 6:**
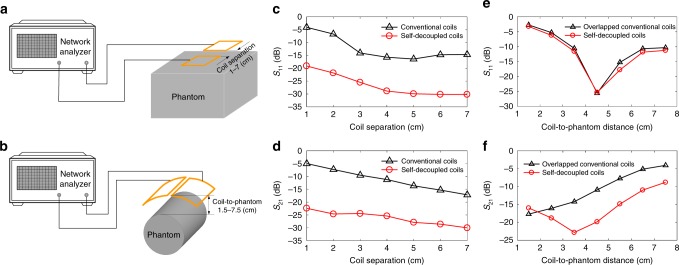
Self-decoupled coil sensitivity to separation and loading. **a** Bench test setup to measure robustness to separation between two adjacent coils. **b** Bench test setup to measure robustness to loading. **c** Measured reflection coefficient (*S*_11_) of conventional and self-decoupled coils vs. coil separation (1 to 7 cm). **d** Measured normalized transmission coefficient (*S*_21_) of conventional and self-decoupled coils versus coil separation (1 to 7 cm). **e** Measured *S*_11_ of conventional overlapped and self-decoupled coils versus coil-to-phantom distances (1.5–7.5 cm). **f** Measured normalized *S*_21_ of conventional overlapped and self-decoupled coils vs. coil-to-phantom distances (1.5–7.5 cm). Compared to conventional coils, the self-decoupled coils had improved robustness to coil separation and similar robustness to loading

Compared to a conventional loop coil, the matching and tuning of a dipole antenna are more sensitive to differences in subject positioning and size due to its higher sensitivity to the parasitic capacitance induced by a load^45^. A self-decoupled coil has a dipole-mode contribution, so a similar concern about robustness in different loading conditions arises. To investigate this, the robustness of self-decoupled coils to different coil separations and loading conditions were evaluated in benchtop experiments and compared to conventional loop coils, using the setup illustrated in Fig. 6a, b.

Figure 6c, d shows the measured *S*_11_ and normalized *S*_21_ of two conventional coils and two self-decoupled coils with different distances between them. S-parameter plots vs. frequency (center 298 MHz, bandwidth 60 MHz) are shown in Supplementary Figure 5. When the two coils were far away from each other, e.g., 7 cm, they were individually well-matched to 50 Ohms with *S*_11_ better than −30 dB (<0.1% power reflection). Although the conventional coils’ coupling was relatively stronger than the self-decoupled coils’ coupling (−17 vs. −29 dB), it was still acceptable with a power cross-talk of ~2%. As the coil-to-coil distance decreased, the coupling increased significantly and was as strong as −4.9 dB for 1 cm separation, leading to resonance peak splitting and an impedance mismatch in the conventional coils (Supplementary Figure 5). For the self-decoupled coils, however, good matching (<−22 dB) and decoupling performance (<−20 dB) were maintained across coil separations. This can be understood by considering that both the electric coupling and magnetic coupling vary similarly as the coil-to-coil distance varies, so they always cancel each other out.

Figure 6e, f shows the measured *S*_11_ and normalized *S*_21_ of two overlapped conventional coils and two self-decoupled coils with different coil-to-phantom distances; loading was higher for shorter distances. S-parameter plots vs. frequency (center 298 MHz, bandwidth 60 MHz) are shown in Supplementary Figure 6. Both the conventional overlapped coils and the self-decoupled coils became mismatched as the coil-to-phantom distance changed. The largest frequency shift and worst impedance matching were 3.6 MHz/−2.8 dB and 7.3 MHz/−3.2 dB for the conventional overlapped and self-decoupled coils, respectively. The normalized *S*_21_ of the conventional overlapped coils ranged from −17.5 to −4.1 dB, while that of self-decoupled coils ranged from −23.7 to −8.8 dB. Although they experienced larger frequency shifts with varied loading (7.3 vs. 3.6 MHz when the coil-to-phantom distance changed from 4.5 to 1.5 cm), the self-decoupled coils’ matching robustness was similar to that of the conventional overlapped coils, and their overall decoupling performance was better, especially for light loading.

### 1×3 and 2×2 self-decoupled arrays

**Fig. 7:**
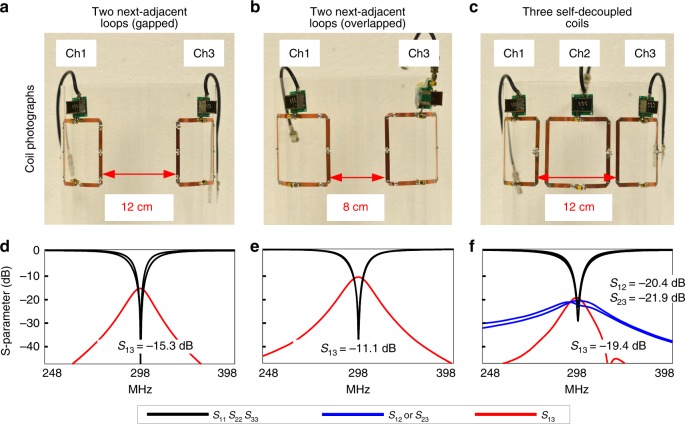
Decoupling of next-adjacent coils and a 1 × 3 self-decoupled array. Arrays were built with two conventional next-adjacent loops with 12 cm separation (**a**; corresponding to next-adjacent loops in a gapped array), two conventional next-adjacent loops with 8 cm separation (**b**; corresponding to next-adjacent loops in an overlapped array) and with three self-decoupled loops (**c**). Measured scattering (S−) parameter plots for the conventional gapped arrays, conventional overlapped array and the self-decoupled array are shown in **d**–**f**, respectively. For the 1 × 3 self-decoupled array, the mutual coupling between next-adjacent elements was −19.4 dB, which is −4.1 dB and −8.3 dB lower than for the conventional loop coil arrays with gapped and overlapped designs, respectively

Cross-talk between non-adjacent (primarily next-adjacent) elements is a challenging issue in decoupling RF arrays, and coupling may exist in multiple dimensions. To evaluate the potential benefits of self-decoupled arrays in terms of coupling between next-adjacent elements, a three-coil self-decoupled array was built (Fig. 7c). For comparison, we also built conventional loop arrays using next-adjacent coil distances corresponding to a gapped design and an overlapped design (Fig. 7a, b). Only two elements were built in the conventional loop arrays since the aim was to evaluate next-adjacent coupling. Figure 7d, e, f plots the arrays’ S-parameters. Even without the influence of the middle coil, the couplings between the next-adjacent coils in the gapped and overlapped designs were −15.3 and −11.1 dB, respectively. The coupling was relatively stronger in the overlapped design because of the slightly larger coil dimensions and smaller coil separations. For the 1 × 3 self-decoupled array the mutual coupling between next-adjacent elements was −19.4 dB, which was −4.1 and −8.3 dB better than the gapped and overlapped designs, respectively.

**Fig. 8:**
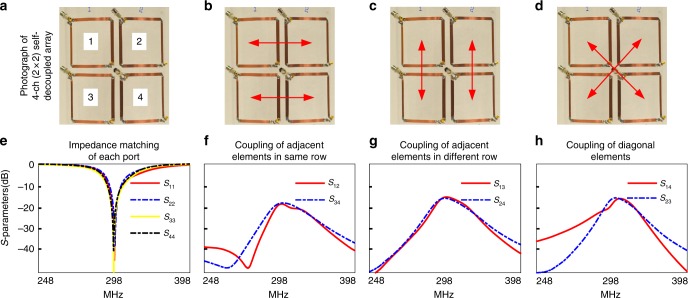
Self-decoupling of a multi-row array. **a** A constructed 2 × 2 self-decoupled array in which the coils’ feed ports are positioned at one corner and the C_mode_ capacitors are positioned at the opposite corner. Red arrows in **b**–**d** illustrated the three kinds of coupling of the 2 × 2 self-decoupled array. The C_mode_ values were chosen to balance decoupling between all elements, and were nearly the same. **e**–**h** Measured scattering parameter plots. The coupling between adjacent elements in the same row, adjacent elements in different rows, and diagonal elements were −17.5 dB, −15.2 dB and −15.4 dB, respectively

The self-decoupled design was further applied to a multi-row array in which three kinds of coupling exist: coupling between neighbouring elements of the same row, coupling between neighbouring elements of different rows and coupling between diagonal elements. A 2 × 2 array was built on a cylindrical former (25 cm diameter), as shown in Fig. 8a. To enable simultaneous decoupling of elements in the same row and different rows (Fig. 8b–d), the C_mode_ capacitors were positioned in the bottom corners of each coil, and the coils were fed in the opposite top corner. Coils in the same row were partly face-to-face, while elements in different rows were not face-to-face. Therefore, magnetic coupling was slightly weaker between elements in different rows, and the optimal C_mode_ value to decouple Coil 1 from Coil 3 was slightly larger (0.48 pF) than the optimal C_mode_ to decouple Coil 1 from Coil 2 (0.37 pF). In the constructed array, the C_mode_ values were chosen to obtain acceptable decoupling between elements in both the same row and in different rows, and Coil 1’s balanced C_mode_ value was 0.44 pF. Diagonal coupling was not considered when tuning C_mode_. With this construction, Fig. 8f, g, h show that the coupling between all elements was better than −15 dB.

### Reducing SAR using multiple C_mode_ capacitors in series and in vivo MR images

**Fig. 9:**
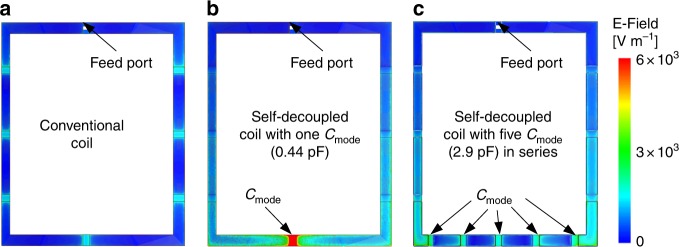
Simulated electric field distributions along the conductor of a conventional coil with eight equally distributed capacitors (**a**, each ~8 pF), a self-decoupled coil with one C_mode_ capacitor (**b**, 0.44 pF) and a self-decoupled coil with five C_mode_ capacitors in series (**c**, each ~2.9 pF). The input power was 1 Watt in each case. The strong electric field near the C_mode_ capacitor was reduced by 72% when it was broken up into five capacitors

Up to this point, only one C_mode_ capacitor was used in self-decoupled coils for simplicity. But this leads to a large electric field across the capacitor’s dielectric and possibly a high local SAR in the subject. Figure 9 shows simulated electric field amplitudes around 10 × 10 cm^2^ conventional and self-decoupled coils with the same input power. Near the C_mode_ capacitor, the electric field of the self-decoupled coil is about 4 times higher than for the conventional coil (7.4 × 10^4^ vs. 1.8 × 10^4^ V m^−1^). This could lead to local temperature rises in the coil housing, but can be addressed by breaking up the C_mode_ capacitor into a capacitance-equivalent set of larger series capacitors^40,46^, or using an equivalent transmission line. With five C_mode_ capacitors, the electric field amplitude was reduced by 72% (2.1 × 10^4^ vs. 7.4 × 10^4^ V m^−1^). This also reduced the 10-gram local SAR by 15%, from 1.66 W Kg^−1^ to 1.44 W Kg^−1^. Therefore, multiple C_mode_ capacitors were used in the self-decoupled coils constructed for in vivo human imaging, described below.

**Fig. 10:**
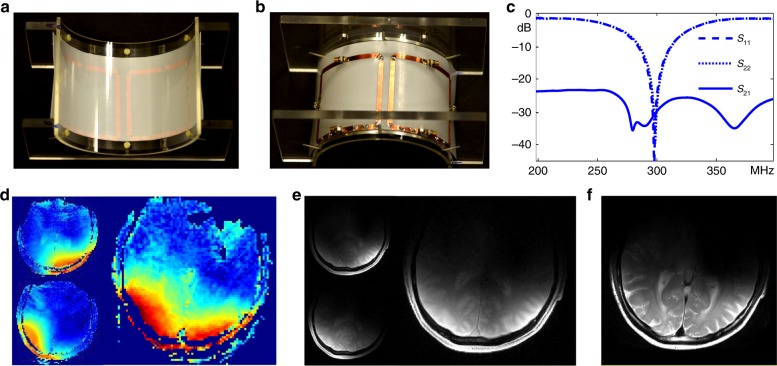
Two-element self-decoupled coil array built for human 7 Tesla MRI. Top (**a**) and bottom (**b**) views of a constructed 2-coil self-decoupled array for human imaging. The coils in this array used five C_mode_ capacitors (each ~2.7 pF) in series along the bottom conductor to reduce the electric field amplitudes near the capacitors. **c** Measured scattering (S−) parameter plots when the array was loaded with a human head. **d** Measured RF transmit field strength (*B*_1_^+^) maps (linear scale) of the two individual coils and their quadrature combination. **e** Measured low-flip-angle gradient echo images of the two individual coils and their quadrature combination. **f** Measured turbo spin echo image. The image is cropped to 16 × 18 cm^2^

Figure 10a, b shows a 2-coil self-decoupled array for human imaging (top view and bottom view). Cable-trap circuits and RF cables were used but are not shown. The coils were initially tuned, matched and decoupled when loaded with a human head, with *S*_11_ and *S*_21_ both better than -30 dB (Fig. 10c). We also note that when the coil was loaded with a human calf, the matching and decoupling remained acceptable without retuning (*S*_11_ and *S*_21_ both better than −18 dB). Figure 10d, e shows measured axial *B*_1_^+^ maps and low-flip-angle gradient recalled echo (GRE) images of the individual coils and both coils driven together. When imaging using both coils, the array was operated in quadrature mode. Each coil exhibited a distinct *B*_1_^+^ map (linear scale) and GRE image, indicating good decoupling performance, which is also consistent with the S-parameter results in Fig. 10c and the phantom results in Fig. 3. Figure 10f shows the turbo spin echo image, which contains no artifacts such as signal voids due to transmit or receive field nulls that could have resulted from coupling between the coils.

## Discussion

We have proposed and validated a novel but simple and effective approach to decoupling loop coils in RF coil arrays. The method requires only intentional tuning of each coils’ capacitance distribution to balance magnetic coupling and electric coupling so that they cancel. It requires no other decoupling treatments or specific coil geometry (overlapping or orthogonality). Even for two very closely spaced loops whose coupling was as strong as −3.6 dB when conventional equally distributed capacitors were used, we showed that excellent isolation could be achieved (−29.3 dB) using self-decoupling while maintaining nearly ideal RF transmit field distributions and power efficiency. We demonstrated that self-decoupled coils are similarly sensitive to loading but have improved robustness to element separation compared to conventional loop coils. Because the electric coupling and magnetic coupling of a self-decoupled coil vary similarly as the coil-to-coil distance varies, they always cancel each other out, which could make self-decoupled coils advantageous in flexible, screen-printed, and size-adjustable arrays^47–49^. In flexible coil designs, the single C_mode_ could instead be replaced by a transmission line to maintain coil flexibility, which may also lower local SAR. Self-decoupling was also effective in decoupling loops from other elements in a mixed loop–dipole array where it is challenging to decouple adjacent (non-overlapping) loop and dipole elements. Another concern for mixed arrays is coupling between adjacent dipole/monopole elements. However, several methods have been described to reduce coupling between closely spaced dipoles/monopoles, such as passive antennas^50,51^ and metamaterials^52^. This report focused on the characterization and validation of self-decoupling itself, and further work is needed to investigate combinations with these methods. Self-decoupling was also effective in decoupling next-adjacent loops and loops in multi-row loop arrays, and could be a key technique in the development of many-element parallel transmission array coils. We expect that decoupling would be improved in a flat self-decoupled array compared to the cylindrical array we built, because the optimal C_mode_ values for decoupling elements in the same row and different rows would be almost the same due to more similar coupling geometries.

In this report we have emphasized the broad applicability and simplicity of self-decoupled designs by building arrays that used only self-decoupling. Although two-dimensional (2-D) and cylindrical arrays are suitable for most applications, close-fitting receive-only three-dimensional (3-D) helmet arrays with 32 or more elements may be preferred to capture maximum SNR in brain imaging. For these arrays, the small size, close spacing and varying neighbour geometries may limit the degree of self-decoupling that can be achieved since coils must be decoupled from several neighbours simultaneously. Further optimization of self-decoupled coils may be needed to address this more complex coupling problem. Alternatively, self-decoupling could be paired with conventional decoupling methods such as overlapping, low-impedance preamplifiers, shielding, and transformers, to address coupling in arrays with complex geometries. In particular, for 3-D receive arrays self-decoupling could combined with preamplifier decoupling to achieve high isolation between all neighbours.

Self-decoupled loop coils are built with unequal capacitance and inductance distributions to intentionally generate electric coupling that cancels magnetic coupling. They are distinct from “loopole” coils^40^, in which vertical conductors are built with unequal capacitance distributions to generate unequal current distributions that approximate transmit- or receive-optimal current patterns at 7 Tesla. However, as shown in Supplementary Figure 7, if a self-decoupled coil is rotated by 90 degrees or fed at its corner, the currents on its two vertical conductors become different so its *B*_1_ field becomes more similar to that of a “loopole” coil^40,46^, which may be helpful to improve transmit or receive efficiency. It should be noted that this may increase coupling with dipole or monopole antennas in the same array. More broadly, self-decoupled coils should enable greater flexibility in RF array design, since they alleviate constraints on element overlap and spacing. As an example, Supplementary Figure 8 shows how decoupling can be made independent of overlapping area using the self-decoupled approach, which provides a new degree of freedom for RF array design to optimize parallel imaging performance and imaging coverage.

Although this work demonstrated the self-decoupled coil concept for 10 × 10 cm^2^ coils at 7 Tesla, it can be applied to other coil sizes and field strengths so long as the magnetic and electric field coupling can be balanced to cancel each other. Like a conventional loop, the resonant frequency of a self-decoupled coil is dominated by its inductance (self-inductance and X_arm_) and capacitance. At lower frequencies and for smaller coils, the X_arm_ impedance needed to tune a self-decoupled coil’s resonant frequency may be an inductor. In particular, if the C_mode_ capacitor required for self-decoupling remained fixed as the coil size decreased, X_arm_ may have a large inductance which could lead to significant power loss. To give some insight on this matter, we simulated a series of square self-decoupled coils with lengths/widths from 10 cm down to 5 cm in 1 cm steps. We found that the C_mode_ increased approximately linearly as the coil size decreased (Supplementary Table 2), and that, for a 5 × 5 cm^2^ self-decoupled coil, the C_mode_ was around 0.8 pF so the required total inductance X_arm_ remained a relatively small 80 nH, which alleviates this concern. We also built a pair of 5 × 5 cm^2^ self-decoupled coils and a pair of 5 × 5 cm^2^ conventional coils. Similar to the 10 × 10 cm^2^ coil, the smaller self-decoupled coil had better decoupling and efficiency than the non-decoupled coils (Supplementary Figure 9). 5 × 5 cm^2^ is smaller than the 9.5 and 6.5 cm coils that have been used in state-of-the-art 32- and 64-coil head arrays, respectively^8^. Supplementary Figure 10 shows a study of self-decoupled coils at 3 Tesla and 1.5 Tesla, where it was found that the ideal *B*_1_^-^ fields can be maintained at 3 Tesla for both 10 × 10 cm^2^ and 20 × 20 cm^2^ coils, but there was an SNR decrease (~20%) for the 10 × 10 cm^2^ coil at 1.5 Tesla due to inductor loss. This loss could be avoided using dielectric materials with high permittivity or meander lines, at the cost of increased manufacturing complexity. The radiation losses of the self-decoupled coils were similar to that of conventional coils, even though they behave more like antennas than conventional coils. This means that most of the coils’ power was directed into the high-permittivity and conductivity samples, which is consistent with results using straight dipole antennas^53^.

## Methods

### Numerical simulation setup

Electromagnetic (EM) simulations were performed using commercially available software (ANSYS Electromagnetics, Canonsburg, PA, USA). As in the experiments, the coils were tuned to 298 MHz (proton Larmor frequency at 7 Tesla) and matched to 50 Ohms (the characteristic impedance of MRI scanner RF chains). Values of all lumped elements were obtained using the EM and RF circuit co-simulation method^54^. During RF circuit optimization, tuning and matching performance were optimized by varying X_arm_ and X_match_ with a built-in module in the RF circuit software (ANSYS Designer), while the decoupling performance was optimized by varying C_mode_ manually. All conductors and lumped elements (capacitors and inductors) were set to be ideal without ohmic loss. The transmit RF fields (*B*_1_^+^) were extracted from the simulations using the relationship: $B_1^ + = (B_{\mathrm{x}} + {\mathrm{i}}B_{\mathrm{y}})/2$^44^, where *B*_x_ and *B*_y_ are the x- and y- components of the magnetic field. Local SAR averaged across 10 grams was calculated using the built-in Fields Calculator module in ANSYS HFSS.

For the loop–loop configuration, the feed port was positioned at one end with one C_mode_ positioned at the opposite end. Two loops (each sized 10 × 10 cm^2^) were mounted on a 25-cm-diameter former and positioned with a 1 cm gap. A cylindrical phantom (conductivity *б* = 0.6 S m^-1^ and relative permittivity *ξ*_r_ = 78) with 15 cm diameter and 20 cm length was placed 4.5 cm below the loops. For the loop–dipole configuration, the loop had one C_mode_ positioned at each end. The dipole had dimensions 23 × 0.75 cm^2^ and loop had dimensions 20 × 9.5 cm^2^; the two were spaced 4 cm apart. A tank phantom with dimensions 35 × 30 × 20 cm^3^ was placed 4.5 cm below the pair (*б* = 0.7 S m^-1^ and *ξ*_r_ = 55). The dipole was shortened using two inductors and was matched using a parallel capacitor^55^.

### Coil construction and bench test setup

The fabricated self-decoupled coils in both loop–loop and loop–dipole configurations had the same geometry and circuit as those in the simulation. The conventional coils used in the comparisons had the same sizes but equally distributed capacitors along their conductors. All coils were constructed with 7.5-mm-wide copper (3 M, Minneapolis, MN). Fixed capacitors (Passive Plus, 111 C Series, Huntington, NY) were used as distributed capacitors and trimmer capacitors (Johanson Manufacturing, 52 H Series, Boonton, NJ) were used for tuning, matching and decoupling. Handmade inductors using American wire gauge (AWG)-18 copper wires were used for X_arm_ impedances when needed. In the simulations, six equally distributed X_arm_ impedances were used for generality. In the constructed coils, only one X_arm_ was used since the total inductance was only around 35 nH. To avoid confusion with the transformer decoupling method which uses a pair of adjacent windings, the inductors in the two coils were placed far away from each other. Bench tests were performed using a calibrated Agilent 5071 C ENA network analyzer. For the loop–loop configuration, a 3-liter cylindrical phantom (15-cm-diameter, with 0.26 g L^−1^ NaCl and 0.125 g L^−1^ NiSO_4_ × 6H_2_O) was used for loading. For the loop–dipole configuration, a 20-liter tank phantom (35 × 30 × 20 cm^3^, with 0.26 g L^−1^ NaCl and 0.125 g L^−1^ NiSO_4_ × 6H_2_O) was used for loading.

### Phantom transmit (*B*_1_^+^) maps and receive sensitivity (*B*_1_^-^) maps

Phantom *B*_1_^+^ maps were acquired with the constructed loop–loop and dipole-loop arrays on a 7 Tesla Philips Achieva whole-body scanner (Philips Healthcare, Best, Netherlands). In all MR experiments, each coil element was measured individually with the other one terminated with 50 Ohms. *B*_1_^+^ maps were measured using the DREAM method with the same input power^56^. The DREAM sequence parameters for the loop–loop configuration (10 × 10 cm^2^) were: field of view (FOV) = 180 × 180 mm^2^, in-plane resolution = 2 × 2 mm^2^, slice thickness = 5 mm, TR = 1000 ms. For the multi-slice *B*_1_^+^ of the 10 × 10 cm^2^ loop coils, the in-plane resolution was set to 1 × 1 mm^2^. The parameters for the loop–dipole configuration were: FOV = 350 × 200 mm^2^, in-plane resolution = 1.8 × 1.8 mm^2^, slice thickness = 5 mm, TR = 1000 ms. To calculate the receive sensitivity (*B*_1_^-^) map of the 5 × 5 cm^2^ loop coils, low-flip-angle gradient-recalled echo (GRE) images were acquired using the following parameters: FOV = 180 × 180 mm^2^, nominal flip angle = 10 degree, in-plane resolution = 3 × 3 mm^2^, slice thickness = 5 mm, TR/TE = 1000/10 ms, bandwidth = 5020 Hz. The *B*_1_^-^ was calculated as Signal Intensity/std(noise)/*|B*_1_^+^|, where std(noise) is the standard deviation of noise map acquired with no RF excitation. Then the *B*_1_^-^ was normalized to the maximum *B*_1_^-^ of the ideal single coils.

### Coil robustness analysis

To evaluate self-decoupled coils’ sensitivity to coil separation, two self-decoupled coils were mounted on a flat acrylic board (thickness 1.2 mm) which had a lift-off of ~4 cm above the large tank phantom. The self-decoupled coils were initially tuned, matched and decoupled when the coils’ distance was 1 cm. Then the distance between the coils (*D*_coil_) was varied from 1 to 7 cm in steps of 1 cm. No re-tuning or re-matching was applied as *D*_coil_ changed. Two conventional coils with the same *D*_coil_ were measured for comparison. The same bench test was conducted for two conventional coils for comparison. During the test, care was taken to make sure the loading condition did not change.

To evaluate self-decoupled coils’ sensitivity to loading, two self-decoupled coils were mounted on a 25-cm-diameter cylindrical former, and were tuned and matched with the 15-cm-diameter cylindrical phantom positioned 4.5 cm below the coils. The coil-to-phantom distance was then increased to 7.5 cm (lighter loading) and decreased to 1.5 cm (heavier loading) in steps of 1 cm. The same bench test was conducted for two overlapped conventional coils for comparison. The overlapped area was optimized when *D*_phantom_ was 4.5 cm, and it was not readjusted when *D*_phantom_ changed. In both cases, *S*_11_ and *S*_22_ were measured to evaluate the matching performance. Note that coils may mismatch when their separation or loading conditions change, so normalized *S*_21_ (defined as $\left| {S_{21}} \right|/\left( {\sqrt {1 - \left| {S_{11}} \right|^2} } \right)$) was used to evaluate the decoupling performance.

### 1×3 array and 2×2 array

The three-coil self-decoupled array was constructed by adding a third coil to the previously described two-coil array. Adding the third coil had little impact on the decoupling performance of the original two coils but slightly changed the resonance frequency of the middle coil, so the C_mode_ capacitor and X_arm_ inductors in the middle coil were retuned manually to achieve isolation and bring its resonant frequency to 298 MHz. We also constructed conventional loop arrays using gapped and overlapping designs as comparisons. Since we focused on evaluating the decoupling between next adjacent elements, only two elements were built in conventional loop arrays. For the gapped array, the two loop elements had the same size (10 × 10 cm^2^) and the same separation (12 cm) as the self-decoupled coils. For the same coverage, the loop elements using an overlapping design had slightly larger size (12 × 10 cm^2^) and closer distance (8 cm). Shielded trap circuits were used for all coils to remove the common current along the cables.

The 2 × 2 array was constructed by adding another row to the two-coil array. As explained above, a suitable dipole mode is needed to cancel out the magnetic coupling and realize self-decoupling. Therefore, each coil in the 2 × 2 array was fed at one corner and the C_mode_ was positioned at the opposite corner to reduce coupling from different rows as well as that from the same row. Due to limited space for the 2 × 2 array, bazooka/sleeve baluns rather than cable traps were used to suppress common-mode current.

### In vivo imaging

The 2-coil self-decoupled array (dimensions 10 × 10 cm^2^) was mounted on an open cylindrical acyclic former (16-cm diameter). To avoid high electric fields near small C_mode_ capacitors, five larger capacitors (four fixed 2.7 pF and one variable ~2.5 pF) were connected in series. The coils were initially tuned, matched and decoupled when loaded with a human head. A healthy male volunteer was imaged using the array on the 7 Tesla whole body scanner. *B*_1_^+^ maps and MR images were acquired with individual coils and both coils, respectively. When imaging using both coils, the array was operated in quadrature mode. The imaging sequence was a low-flip-angle GRE sequence with parameters: FOV = 220 × 220 mm^2^, nominal flip angle = 10 degrees, TR/TE = 1000/1.98 ms, in-plane resolution = 1 × 1 mm^2^, slice thickness = 5 mm, bandwidth = 887 Hz pixel^-1^, number of averages = 1. Turbo spin echo (TSE) images were also acquired with the parameters: FOV = 220 × 178 mm^2^, refocusing flip angle/flip angle = 120/90 degrees, TR/TE = 3000/76 ms, in-plane resolution = 0.5 × 0.5 mm^2^, slice thickness = 5 mm, bandwidth = 243 Hz pixel^-1^, TSE factor = 15, number of averages = 1. To achieve the same *B*_1_^+^ in this area, the required input power of the 2-coil self-decoupled array was 73% lower than for a commercial volume transmit array (28 cm diameter, Nova Medical Inc., Wilmington, MA). The human experimental procedures were approved by the local institutional review board of Vanderbilt University, and informed written consent was obtained from the participant.

### Data Availability

The data that support the findings of this study are available from the corresponding author upon request.

## Electronic supplementary material

Supplementary Information

Peer Review file
