# Supplementary material for: Self-decoupled radiofrequency coils for magnetic resonance imaging
Source: Nat Commun. 2018 Aug 28;9:3481. doi: 10.1038/s41467-018-05585-8 (PMC6113296; doi:10.1038/s41467-018-05585-8)
Supplement: Supplementary file 1 — Supplementary Information [file 41467_2018_5585_MOESM1_ESM.pdf]

Supplementary Information

Self-decoupled radiofrequency coils for magnetic resonance imaging

Yan *et al.*

The Supplementary Information includes 10 Supplementary Figures and 2 Supplementary Tables.

C_{mode} (pF)	f_{odd} (MHz)	f_{even} (MHz)	f_m (MHz)	K_m	K_e	K_{total}
**8.00**	308.2	289.2	195.0	-0.110	0.047	-0.063
**6.00**	308.2	289.2	196.0	-0.110	0.048	-0.063
**4.00**	308.4	289.2	199.6	-0.114	0.052	-0.063
**2.00**	308.0	289.6	202.0	-0.112	0.052	-0.060
**1.00**	302.0	291.2	241.0	-0.109	0.071	-0.037
**0.80**	300.6	292.2	253.2	-0.108	0.078	-0.030
**0.60**	298.6	294.0	270.8	-0.098	0.082	-0.017
**0.30**	293.8	298.8	323.0	-0.087	0.103	0.016
**0.20**	291.8	300.8	350.0	-0.076	0.106	0.029
**0.10**	289.4	303.2	386.0	-0.067	0.112	0.046
**0.01**	287.0	306.2	438.0	-0.055	0.119	0.064

Supplementary Table 1 Calculated coupling coefficients using full-wave electromagnetic simulations. The coils had the same size and geometry as those in Figure 2. First, a single coil was tuned to the Larmor frequency (f_0=298 MHz). Then its resonance frequencies in odd mode (f_{odd}) and even mode (f_{even}) were obtained by inserting an electric wall and a magnetic wall (Hong & Lancaster, IEEE Trans. Microw. Theory Techn. 44, 2099-2109, 1996), respectively. f_m is the transmission zero frequency in the presence of another identical coil. Finally, the magnetic (K_m, loop-mode) coupling coefficient, the electric (K_e, dipole-mode) coupling coefficient and the total coupling coefficient (K_{total}) were calculated based on the following equations from Chu *et al.* (IEEE Trans. Microw. Theory Techn. 56, 431-439, 2008):

$$|K_m| = \frac{1}{2}\left(\frac{f_{odd}^2-f_0^2}{f_{odd}^2-f_m^2} + \frac{f_{even}^2-f_0^2}{f_m^2-f_{even}^2}\right) \quad (1)$$

$$|K_e| = \frac{f_m^2}{2f_0^2}\left(\frac{f_0^2-f_{odd}^2}{f_m^2-f_{odd}^2} + \frac{f_0^2-f_{even}^2}{f_{even}^2-f_m^2}\right) \quad (2)$$

$$K_{total} = \frac{K_m+K_e}{1+K_mK_e} \quad (3)$$

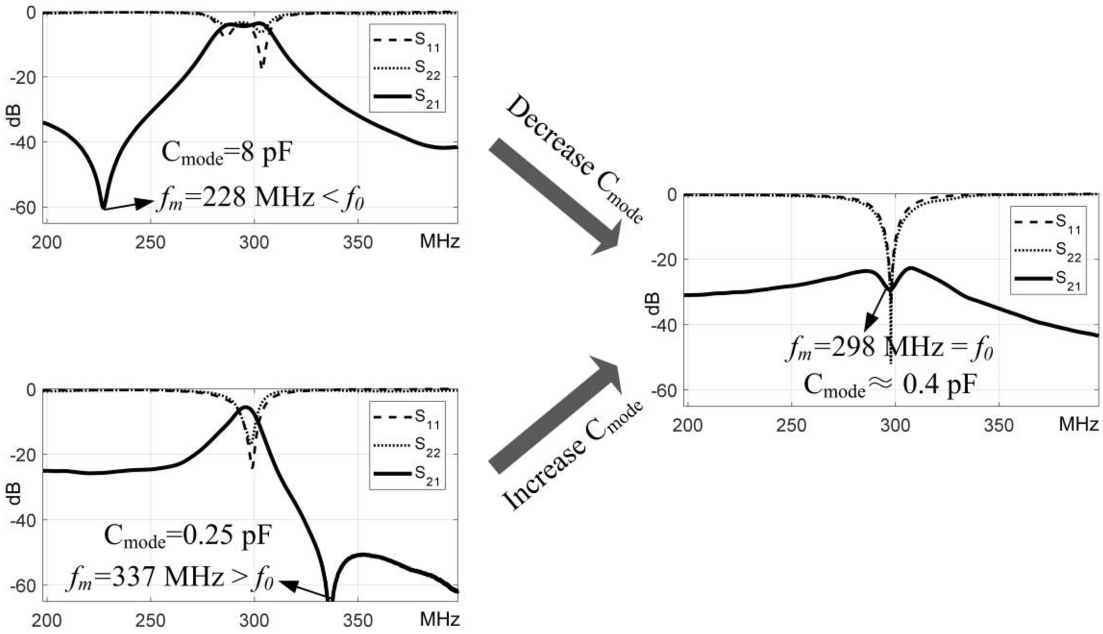

Supplementary Figure 1 Illustration of how to tune C_{mode} to achieve self-decoupling based on bench measurements of S_{21}. When f_m is lower (**left, top**) or higher (**left, bottom**) than the desired resonance frequency f_0, C_{mode} needs to be decreased or increased, respectively, to bring f_m to f_0 and achieve the best decoupling performance.

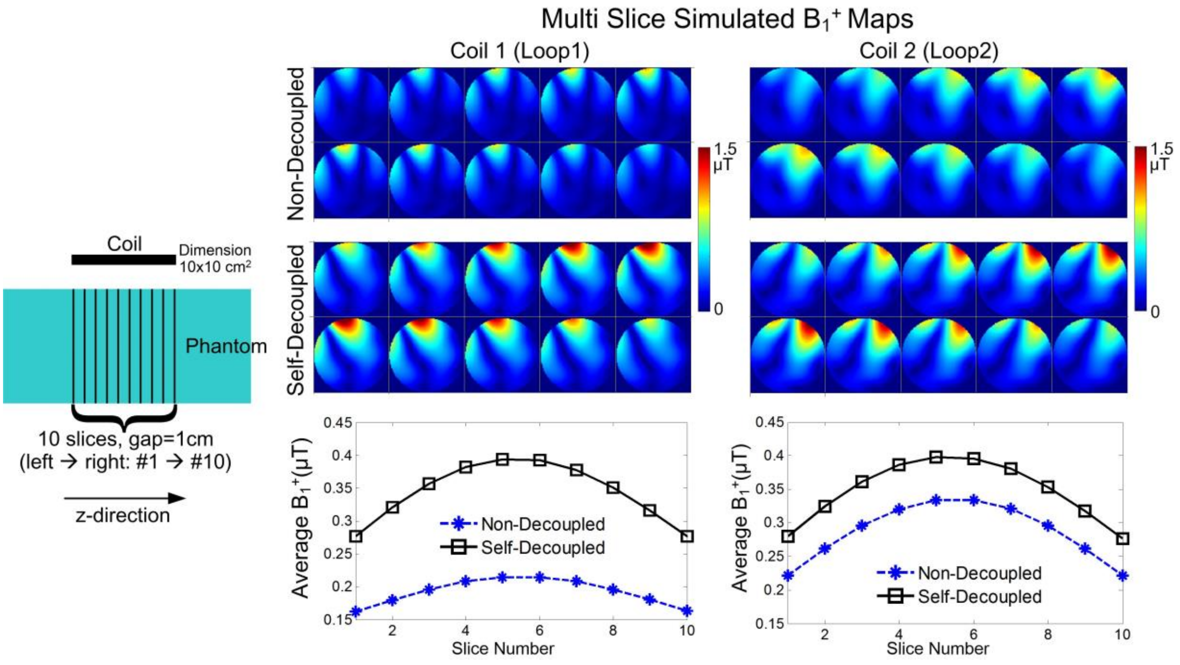

Supplementary Figure 2 Simulated multi-slice axial B_1^+ maps of two non-decoupled conventional coils (top row) and two self-decoupled coils (middle row) in a loop-loop configuration. The same input power (1 Watt) was used for all measurements. The bottom row plots the average B_1^+ in each slice. Number of slices = 10, Slice gap = 1cm. Although the current is non-uniform in self-decoupled coils (stronger near the feed port and weaker near the C_{mode} capacitor), the slice-by-slice B_1^+ maps decay similarly to non-decoupled coils' maps. This can be understood by considering that the $B_1^+=(B_x+iB_y)/2$ field is mainly produced by the current on the vertical conductor segments for this square coil, where the current distribution is relatively uniform.

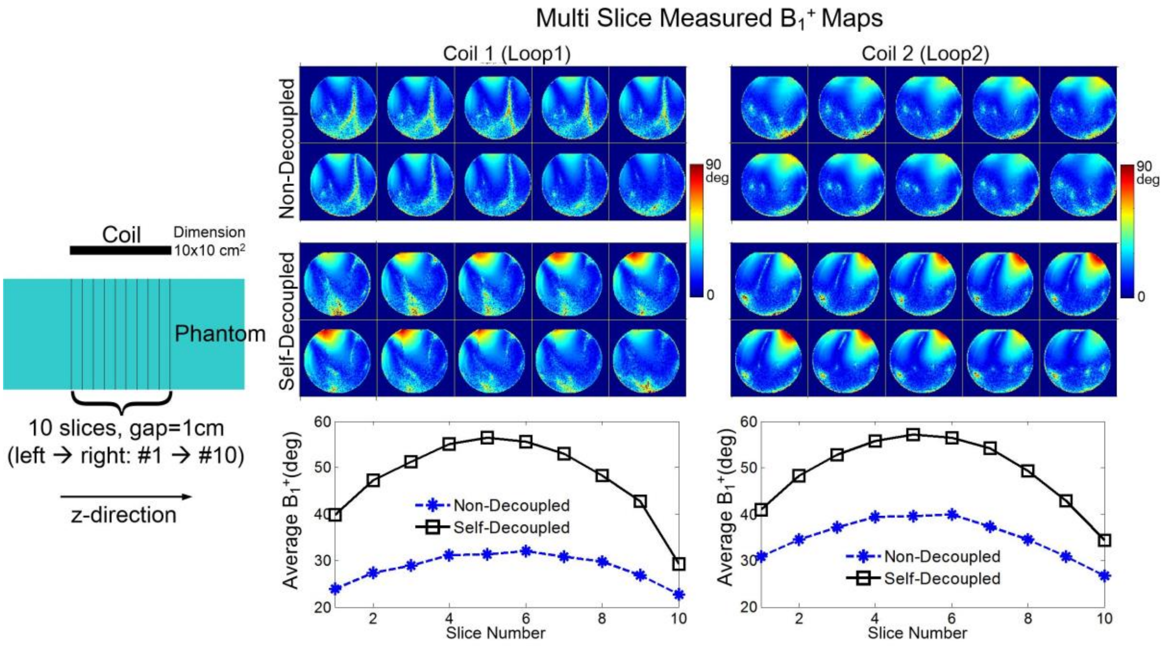

Supplementary Figure 3 Measured multi-slice axial B_1^+ maps of two non-decoupled conventional coils (top row) and two self-decoupled coils (middle row) in a loop-loop configuration. The same input power was used for all measurements. The bottom row plots the average B_1^+ in each slice. Number of slices = 10, Slice gap = 1 cm. The overall experimental results are consistent with the simulated B_1^+ maps in Supplementary Figure 2. The measured B_1^+ maps are not as uniform along the z-direction as the simulated maps, which is because (due to its small value) only one X_{arm} inductor was used in the constructed coils while six X_{arm} were used in the simulated coils.

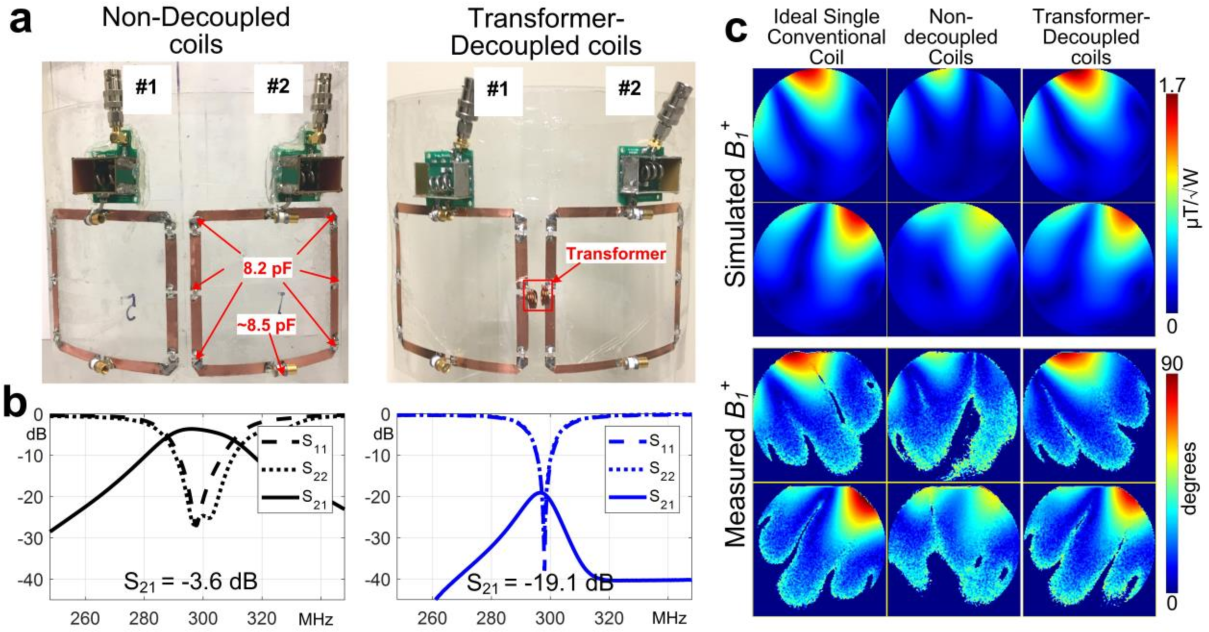

Supplementary Figure 4 Comparison of two-loop non-decoupled conventional coils and transformer-decouple coils. **a)** Constructed two-element non-decoupled conventional **(left)** and transformer-decoupled **(right)** coil arrays, with the same dimensions as the simulated coils in Figure 2. **b)** Measured S-parameter plots of the non-decoupled conventional **(left)** and transformer-decoupled coils **(right)**. **c)** Simulated and measured axial RF transmit field strength (B_1^+) maps of ideal single conventional coils, the two non-decoupled conventional loops and the two transformer-decoupled coils.

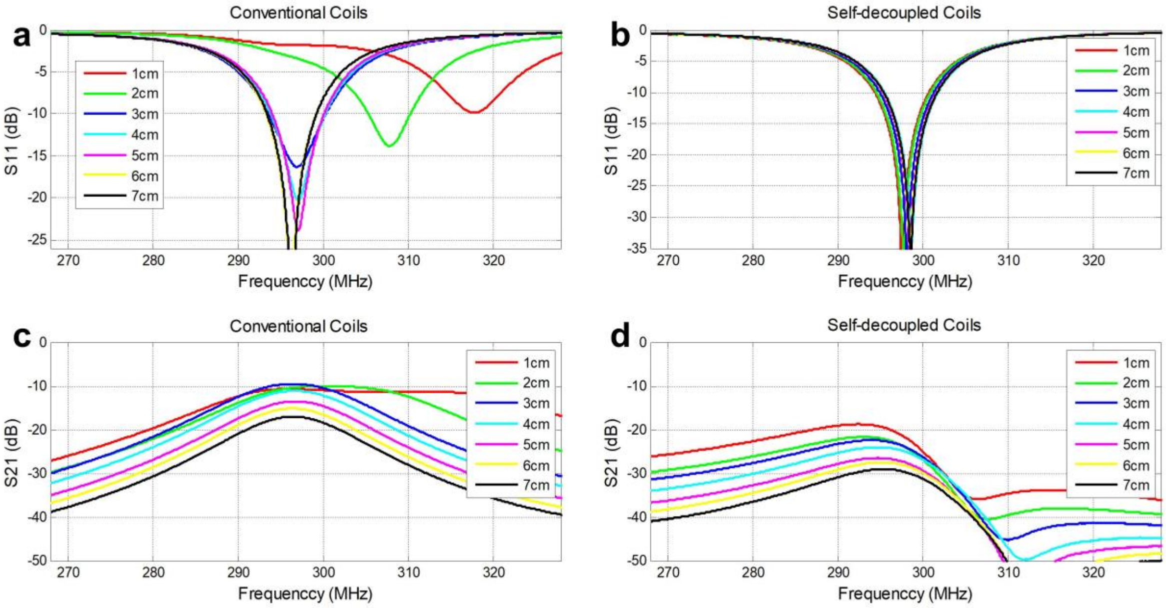

Supplementary Figure 5 Measured S-parameter plots versus frequency with different coil separations (D_{coil}, from 1 cm to 7 cm with steps of 1 cm). **a)** S_{11} plots (matching performance) of two conventional coils. **b)** S_{11} plots of two self-decoupled coils. **c)** S_{21} plots (decoupling performance) of two conventional coils. **d)** S_{21} plots of two self-decoupled coils. The conventional coils' performance depends strongly on D_{coil}. For D_{coil} less than 3 cm, the strong coupling caused resonant peak splitting and impedance mismatch. For the self-decoupled coils, however, excellent matching (<-22 dB) and decoupling performance (<-20 dB) were maintained as D_{coil} changed from 7 cm to 1 cm.

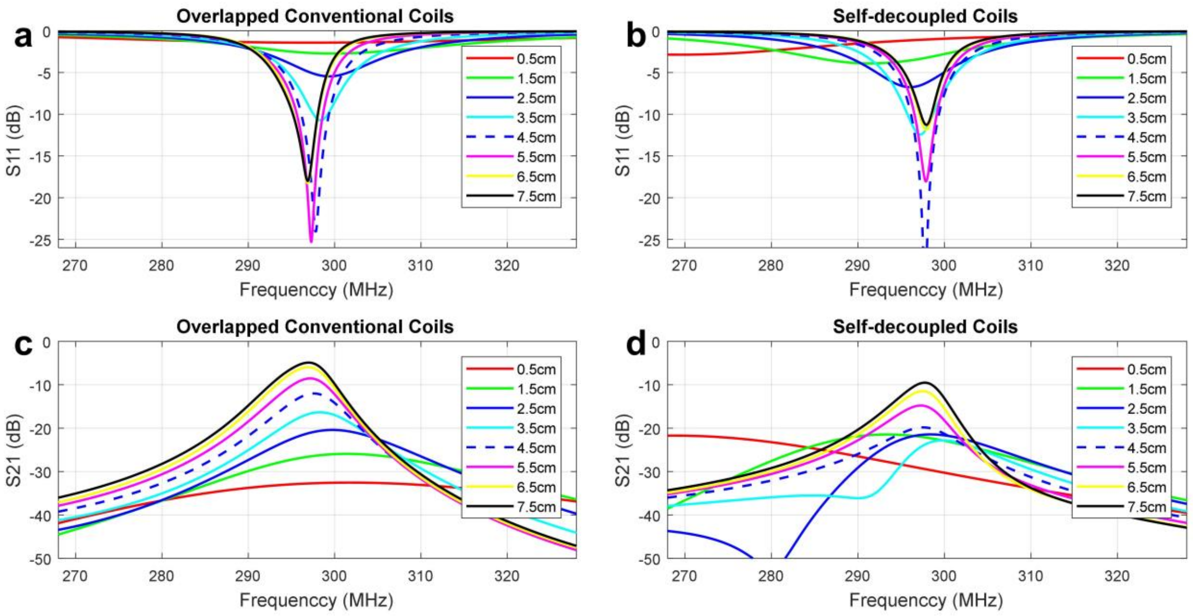

Supplementary Figure 6 Measured S-parameter plots versus frequency with different coil-to-phantom distances (D_{phantom}, 1.5 cm to 7.5 cm in 1 cm steps). The coils were initially tuned, matched and decoupled when D_{phantom} = 4.5 cm, and were not readjusted for other D_{phantom}. **a)** S_{11} plots (matching performance) of two overlapped conventional coils. **b)** S_{11} plots of two self-decoupled coils. **c)** S_{21} plots (decoupling performance) of two overlapped conventional coils. **d)** S_{21} plots of two self-decoupled coils. Compared to the conventional coil, the self-decoupled coil has similar matching robustness but more obvious resonance frequency shift. The decoupling performance of self-decoupled coils is overall better compared to overlapped conventional coils, especially in the light loading (large phantom-coil distance) case.

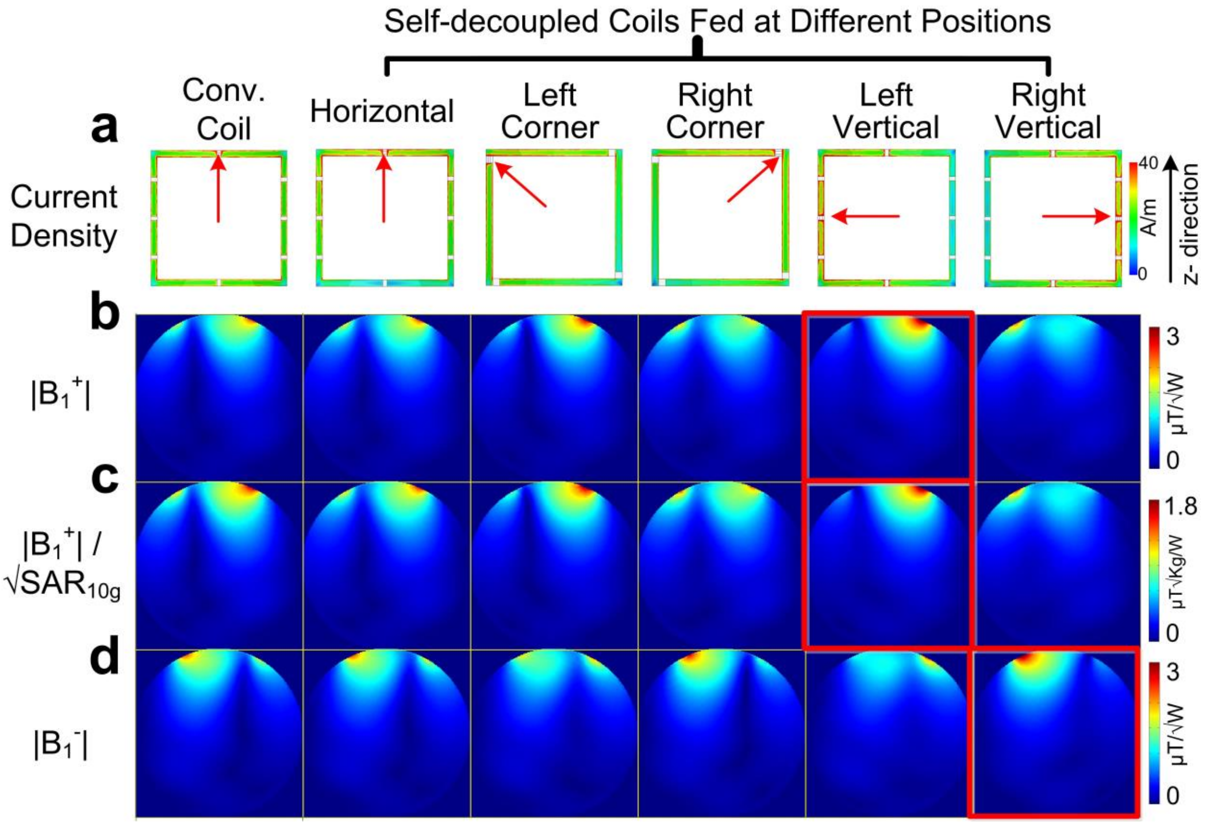

Supplementary Figure 7 Simulated axial B_1 maps of a conventional coil (uniform current distribution) and self-decoupled coils fed at different positions, as indicated by the red arrows in Supplementary Figure 7a. All coils were 10×10 cm^2 in size and were placed 1 cm away from a cylindrical phantom (diameter 20 cm, length 30 cm, σ = 0.6 S m^{-1} and ξ_r = 78). When fed in its vertical conductor, the self-decoupled coil exhibited "loopole-type" B_1 patterns, which can increase either B_1^+ or B_1^- at the expense of decreasing the other, which is consistent with previous work. In this simulation, the "loopole-type" B_1 patterns had notable improvements (average 18%) in either transmit efficiency or receive sensitivity (red boxes).

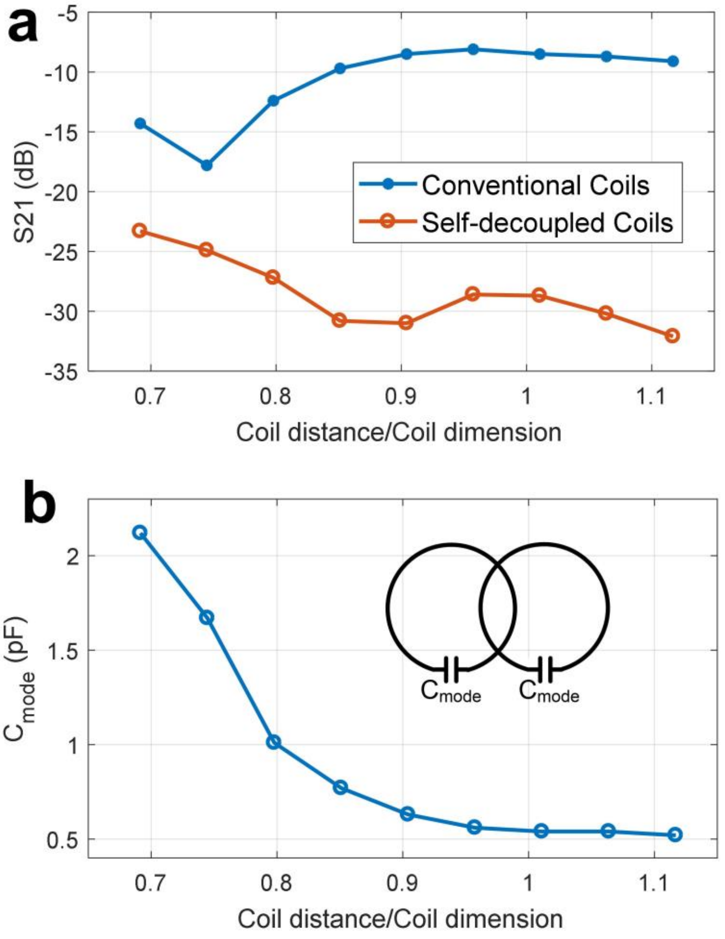

Supplementary Figure 8 Decoupling capability versus different coil separations. **a)** Simulated S_{21} of a pair of self-decoupled coils and a pair of conventional coils as a function of the coils' center-to-center distance (i.e., overlapping area). For the conventional coils, a critical overlapping area was required for decoupling. However, the self-decoupled coils maintained excellent decoupling performance over a wide range of overlapping areas. **b)** C_{mode} as a function of coil distance. Each circular loop had a dimension of 10 × 10 cm^2 and was placed 2 cm away from a tank phantom (dimension 40 × 30 × 20 cm^3, σ = 0.7 S m^{-1} and ξ_r = 55).

Coil size (cm^2)	C_{mode} (MHz)	Total X_{arm} (nH)
**10×10**	0.44	33.6
**9×9**	0.50	49.2
**8×8**	0.57	60.4
**7×7**	0.65	70.1
**6×6**	0.73	77.7
**5×5**	0.82	82.2

Supplementary Table 2 Simulated values of C_{mode} and total X_{arm} of self-decoupled coils across a range of dimensions (square loop, from 10 × 10 cm^2 to 5 × 5 cm^2). All coils are tuned to 298 MHz (Larmor frequency at 7 Tesla) and matched to 50 Ohms. The C_{mode} values increased approximately linearly as the coil size decreased.

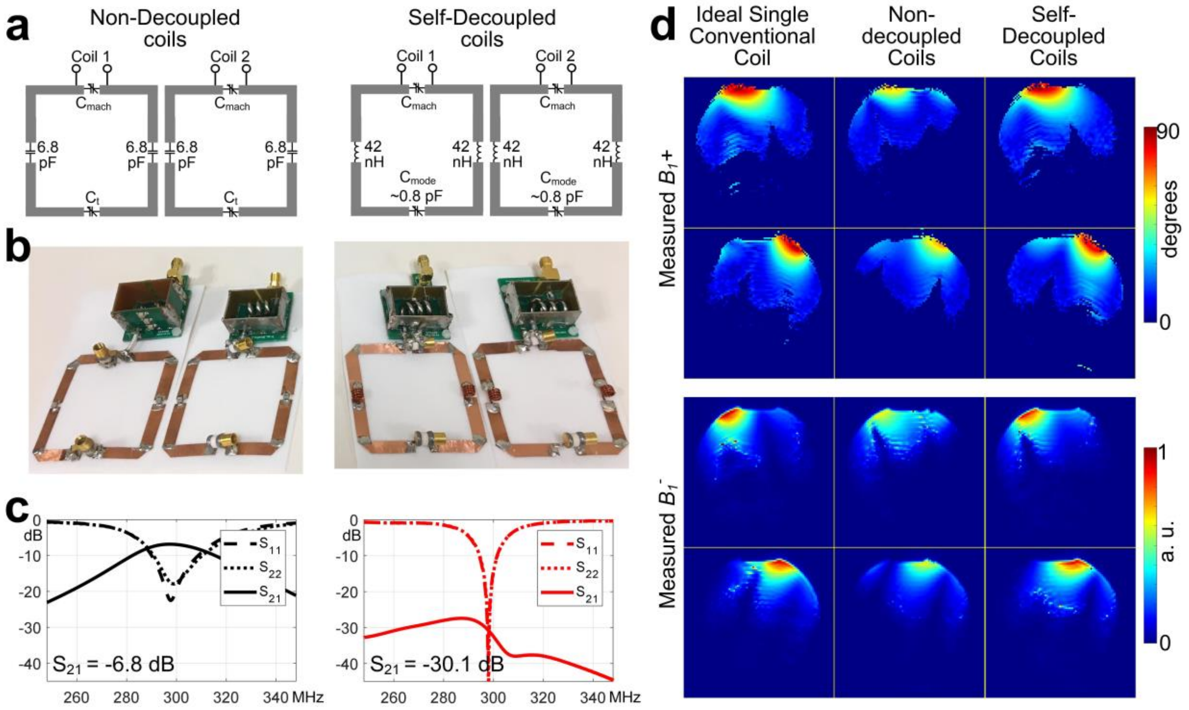

Supplementary Figure 9 Small (5 × 5 cm^2) two-loop coil arrays. **a)** Diagrams of two-element conventional **(left)** and self-decoupled **(right)** coil arrays. **b)** Constructed two-element conventional **(left)** and self-decoupled **(right)** coil arrays. **c)** Measured S-parameter plots of the conventional **(left)** and self-decoupled coils **(right)**. **d)** Measured B_1^+ and normalized B_1^- maps of ideal single coils, two conventional loops and two self-decoupled coils in a transverse slice. Compared to the non-decoupled coils, Loop 1 and Loop 2 of the self-decoupled coils had 37% and 21% higher B_1^+, and 23% and 31% higher B_1^-.

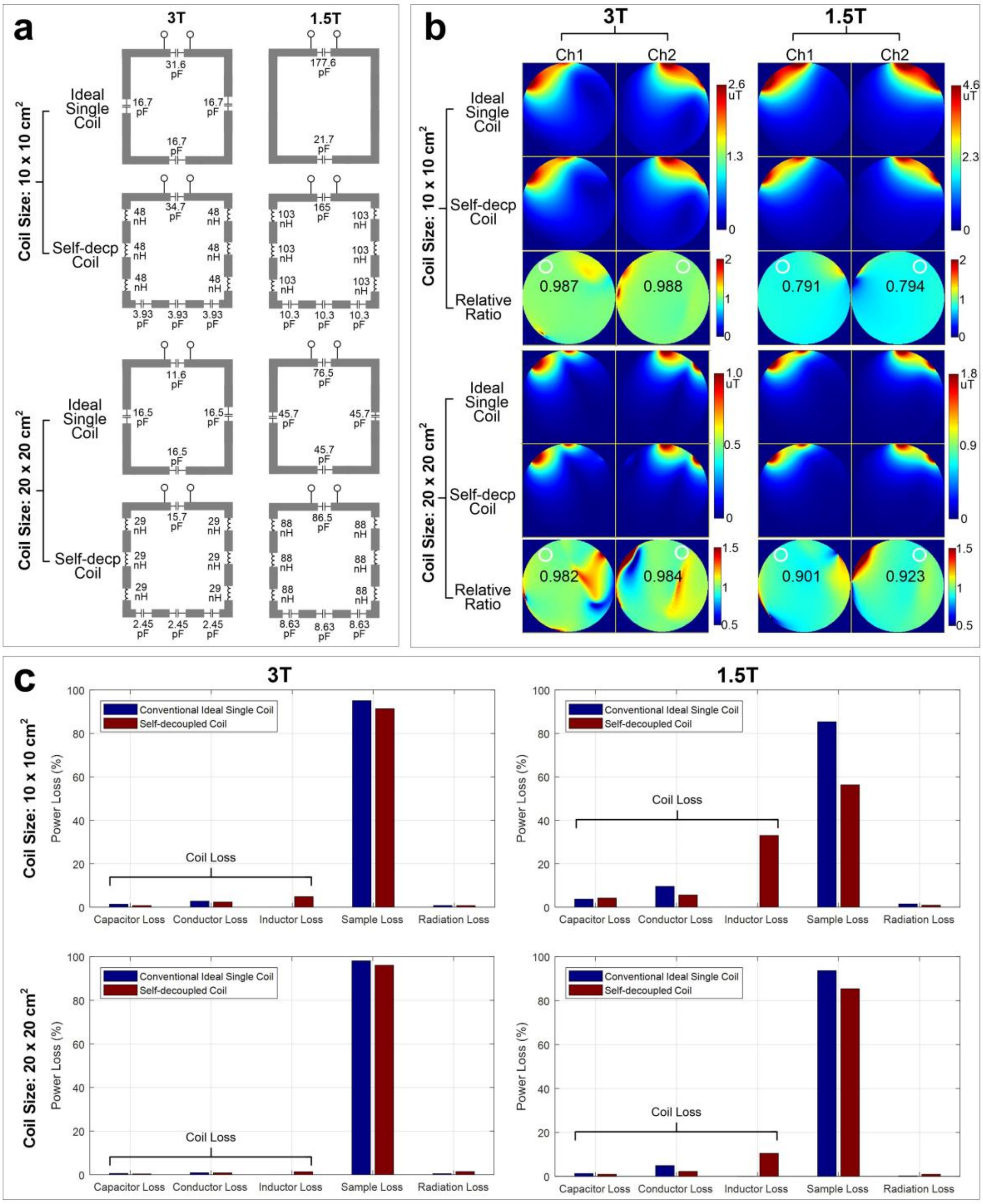

Supplementary Figure 10 Self-decoupled coils at 1.5 Tesla and 3 Tesla. **a)** Schematic of a single conventional coil with equal capacitance distribution and a single coil from a two-element self-decoupled array at 3 Tesla and 1.5 Tesla. Coils with two different dimensions (10 × 10 cm^2 and 20 × 20 cm^2) were simulated. All coils were wrapped around a cylindrical phantom (σ = 0.6 S m^{-1} and ξ_r = 78) with a separation of 1 cm. The diameters of the cylindrical phantoms were 20 cm and 40 cm for the 10 × 10 cm^2 coil and the 20 × 20 cm^2 coil,

respectively. Coil conductors were modeled as copper sheets with a conductivity of 5.8 × 10^7 S m^{-1}, and capacitors and inductors were modeled as lossy components considering series resistance. The quality (Q-) factors of the capacitors were between 1000 to 2000 based on datasheets of commercial high-Q non-magnetic capacitors (Passive Plus, 111C Series, Huntington, NY), and the Q-factors of the inductors were set to 250. As in the real case, the coil impedances were well matched to 50 Ohms, with S_{11}'s less than -30 dB. The isolation between the pair of self-decoupled coils was less than -25 dB. In all simulations, the input power was set to 1 Watt. **b)** Central axial receive sensitivity (B_1^-) maps for different coil sizes at 3T and 1.5T. Compared to the ideal single coil without the presence of the other coil, the receive sensitivity of the self-decoupled coil was maintained at 3T, with a decrease < 2%. At 1.5 T, however, the receive sensitivity loss is larger, up to 21% for a 10 × 10 cm^2 coil. **c)** Analysis of power loss. The power losses were calculated by integrating the surface loss density or volume loss density using a built-in function in the simulation software (ANSYS HFSS, Canonsburg, PA, USA). A significant amount of power was lost in the inductors of the self-decoupled coils (X_{arm}) at 1.5 T, partly because the required inductor had a large value, and partly because coil losses are generally larger at low fields. We note that the conductor loss of the self-decoupled coil is slightly smaller than the conventional coil due to its high-impedance structure. The power loss results are consistent with the results in Supplementary Figure 10b, specifically that higher sample loss leads to higher receive sensitivity.
