# Supplementary material for: Self-decoupled radiofrequency coils for magnetic resonance imaging
Source: Nat Commun. 2018 Aug 28;9:3481. doi: 10.1038/s41467-018-05585-8 (PMC6113296; doi:10.1038/s41467-018-05585-8)
Supplement: Supplementary file 2 — Peer Review file [file 41467_2018_5585_MOESM2_ESM.pdf]

Reviewers' comments:

Reviewer #1 (Remarks to the Author):

The authors present a method that intrinsically decouples a loop antenna from a neighbouring loop or dipole by equating the current density in the loop and dipole modes of the coil element. Self-decoupled RF coils exhibit excellent decoupling between adjacent and next-nearest-neighbour elements, with robustness to coil loading and the distance between coils.

Self-decoupled RF coils appear to be a clever means to decouple RF coils without the use of electrical circuits or geometric overlap. This is the first time the difference in current density of loop-mode and dipole-mode coupling has been exploited for this purpose. This technique is certainly original for decoupling RF coils, and is a valuable extension to the loophole introduced by Wiggins et al. This should be of great interest to MRI coil designers, both in academia and industry.

The manuscript is scientifically sound, with no major flaws. The authors address the majority of relevant questions (some further questions are detailed below) regarding a new decoupling scheme-B1+ efficiency, B1+ profile, dependency on load and distance, next-nearest-neighbour coupling, 2D arrays, and local SAR. The methods used to evaluate the decoupling scheme are of high quality and presented in a clear and concise manner. Some additional comments on how to determine Cmode for a large array would be useful to the reader in order to reproduce and implement this decoupling scheme. The abstract and introduction are sufficiently broad and should be accessible to a wide audience. The conclusions are in keeping with the results, although the utility for receive coils may be overstated, as discussed below.

For transmit coils, where geometries are typically simpler than receive coils and often cylindrical, self-decoupled coils should provide a practical means to substantially increase the isolation between coil elements of a loop coil. The ability to produce high isolation between transmit coils, without the necessity of electrical connections or geometric overlap, would be of great use and provide many unique possibilities for transmit coil design. Decoupling transmit coils is still an active area of investigation, and self-decoupling coils presents a potentially large step forward.

When applying the technique to receive coils, the technique is perhaps less useful, for several reasons. First, as geometries become complex, such as for a 32-channel head coil, a single loop will be located at many different angles with its neighbours, thereby causing the designer to compromise in their choice of Cmode; Figure 1 shows a sharp increase in coupling with small changes to Cmode. The authors present a 2x2 array, with still a relatively simple geometry, and yet the decoupling reduces to approximately -15 dB. Although this is still good, it seems as though this would become worse for an increasingly complex geometry where a greater degree of compromise is required when determining Cmode. Furthermore, the decoupling is still not sufficient to make the use of low-input-impedance preamplifiers unnecessary, so I am wary that the use of self-decoupled coils will not change the way receive coils are designed.

A second major drawback for receive coils is the seeming reliance on coil diameters to be relatively large to prevent the need for large, lossy inductors. As mentioned by the authors, meanders could be used, but the space requirements for a high-density receive coil may make this impractical. They also tend to become more lossy unless careful simulation of the meanders is conducted. This is less of an issue for transmit only coils.

Specific comments/suggestions:

1. It could be useful to comment on the practical implementation of this procedure. The authors show in Figure 7 that the inclusion of the third coil element changes the optimal Cmode. Given the dependence of Cmode on neighbouring coils, would the entire array be simulated, then Cmode

determined from simulation? Or is there a practical method for adjusting Cmode on the bench for a large array of coils?
2. For the 2x2 array, the authors state that Cmode was chosen as a compromise for the three coil-combinations causing coupling. If Cmode was not chosen as a compromise, but chosen as the optimal value for each of the three coil combinations, what would the range in Cmode be? Given Figure 1, there seems to be a large increase in coupling with small changes in Cmode. For a head coil that has a spherical-like shape, would this compromise in Cmode render the method unusable? Certainly, for cylindrical coils this is much less of a problem and works quite well.
3. One measurement that is missing is the comparison B1+ efficiency (or B1- sensitivity) of a single conventional coil to a single self-decoupled coil. It may be sufficient to reference Lakshmanan et al. (Ref. 31) for this; however, it is necessary to show that B1 of s asingle coil is not sacrificed (excluding the effects of coupling) by manipulating the current density in this manner.
4. To compare the B1+ efficiency of two coupled conventional coils with two self-decoupling coils is a bit misleading. Some means (either geometric overlap, capacitive or geometric decoupling, etc.) would always be used to decouple conventional coils, which would improve the B1+ efficiency.
5. Why is the optimal experimental coupling between a dipole and a self-decoupling loop only -14.8 dB? If the loop-mode and dipole-mode currents cancel, shouldn't the isolation between the two elements be better? Or is this residual coupling due to resistive coupling through the conductive sample?
6. For Figure 6, please explain how S21 is normalized, as this was not clear in the text.
7. The authors state that self-decoupling coils may become less efficient for smaller coil diameters. Could the authors provide a lower bound for where they feel self-decoupling coils will be useful.
8. In Figure S2, why is there considerably more asymmetry in B1+ as a function of slice number versus the simulation?
9. References to alternative decoupling schemes should be expanded or modified, given this is the primary focus of the manuscript. For decoupling networks, Lee et al. predates the references provided. Pinkerton et al. is the seminal US patent and decoupling method used in commercial scanners (builds on Lee). Methods such as resonant-inductive decoupling (Avdievich et al.) and Cartesian feedback (Hoult et al.) should also be included:
" Lee RF, Giaquinto RO, Hardy CJ. Coupling and decoupling theory and its application to the MRI phased array. Magn Reson Med 2002;48(1):203-213.
" Pinkerton RG, Barberi EA, Menon RS. Noise properties of a NMR transceiver array coil. J. Magn. Reson. 2004; 171(1):151-156.
" Avdievich NI, Pan JW, Hetherington HP. Resonant inductive decoupling (RID) for transceiver arrays to compensate for both reactive and resistive components of the mutual impedance. NMR Biomed 2013;26(11):1547-1554.
" Hoult DI, Kolansky G, Kripiakevich D, King SB. The NMR multi-transmit phased array: A Cartesian feedback approach. J Magn Reson 2004;171(1):64-70.

Ravi S. Menon, Ph.D.
Kyle M. Gilbert, Ph.D.

Reviewer #2 (Remarks to the Author):

Overall impressions:
This manuscript describes an innovative radiofrequency coil design for magnetic resonance imaging and spectroscopy, addressing the important practical challenge of coupling among elements of a coil array. The self-decoupling design is in principle fairly simple to implement, as a perturbation on traditional coil designs, lending it widespread potential practical value and day-to-day impact. From this point of view, I think that the manuscript would be received with interest and enthusiasm by a broad audience. There are, however, several factors which reduce my

enthusiasm for the paper in its current form, including

1. The absence of a solid theoretical explanation of the observed decoupling effects, which might help to provide insight into the range of conditions over which favorable effects might be expected.
• The authors assert that currents induced by a neighboring coil in the loop-like and dipole-like virtual components of their self-decoupled design are oppositely directed but otherwise equivalent in distribution. This is undoubtedly a convenient property, but it is not at all obvious why this should be the case. In fact, though the simulated current distributions in loop-mode and dipole-mode configurations (e.g. in Fig. 2 and Fig. 4) are indeed oppositely directed, they do not appear to share the same distribution around the coil, and they would therefore NOT cancel one another. The explanation for self-decoupling, then, is not fully satisfying. One might argue, fairly enough, that the experimental results show value regardless of how convincing the theory may be. However, in the absence of a solid theoretical backdrop, it is difficult to know how generalizable the self-decoupling effect may be in practice. One suggestion might be to separate the self-decoupled coil into its loop-like and dipole-like components, and to perform a separate simulation with each component as a passive second coil paired with the same transmitting coil. I must confess that it is difficult to imagine, based on fundamental symmetries, that the resulting induced current contributions would ever be able to cancel one another, so perhaps there is some fallacy in the superposition argument which remains to be exposed. The fact that a transmitting dipole appears to be decoupled just as well as a transmitting loop from the self-decoupled structure is interesting, and also puzzling. How can the geometry of the transmitting structure not matter? Is the properly-tuned self-decoupled structure somehow incapable of carrying a current at all unless it is imposed at the transmit port? Simulations like those in Figs. 2 and 4, but with Cmode tuned to the self-decoupled condition, would be interesting to see. Or is the self-decoupled structure in fact a high-impedance structure? One would expect this to result in a low transmit efficiency, however, which does not appear to be the case. Color me curious, but confused.

2. Certain considerations which might make the self-decoupled design cumbersome in practice, including
• The need to break up the Cmode capacitor to avoid excessive local SAR, resulting in a comparatively large number of closely-spaced lumped elements. This might prove a challenge for flexible coil designs, and might also result in undesired losses for transmit and/or receive efficiency.
• The need to adjust the position of the Cmode capacitors depending upon the desired decoupling topology. This might be a limitation for the general case of many-element arrays, and it might also influence the transmit and receive sensitivity patterns, which the authors acknowledge will depend upon the orientation of the coils and their ports.

3. The absence of considerations of receive performance
• Although the transmit case is arguably the most critical case calling for improved decoupling, receive arrays have a much higher volume of use than transmit arrays, and therefore improvements in receive performance would have a higher overall impact. Reciprocity might lead one to expect receive performance gains that are similar to the gains explored in the transmit setting; however, the devil is often in the details when it comes to RF engineering, and the absence of a clear theoretical backdrop makes it harder than it might otherwise be to rely upon simple intuitions. For example, how would the presence of a low-input-impedance preamplifier affect the decoupling conditions? What about losses in the lumped elements of the self-decoupled design? A detailed exploration of receive effects could legitimately be argued to be beyond the scope of this manuscript, but at least some commentary on expected effects would be welcome.

On the whole, the manuscript represents a potentially valuable contribution to literature, whose full impact is, however, somewhat difficult to judge without more information.

Additional detailed comments:

• P. 4, lines 55-56: “When receiving RF signals from the body, coupling leads to noise amplification and limits the degree to which a scan can be accelerated using parallel imaging.” This is not entirely true. It has been shown that SNR-optimizing reconstructions accounting for noise correlations can undo the effects of moderate degrees of linear inductive coupling. It is only in more extreme cases that the parallel imaging performance, and overall electrical performance, of coupled coils may be degraded. A slightly more nuanced statement might be appropriate.
• Figure 3 and Supplementary Figure 1: It is not entirely clear why the B1+ profile in the self-decoupled case is so close to that in the ideal uncoupled case with uniform currents. The authors make the argument that only two legs of the coil loop are important for B1+ generation, but this is true only in certain coil and slice orientations. A change in current distribution must affect the sensitivity profile in general. Here some simulations of the current distribution in the self-decoupled case – rather than the loop-mode and the dipole-mode cases shown in Figures 2 and 4 – could be helpful.
• Figure 4: As mentioned earlier, it is interesting that the self-decoupled coils show similar decoupling behavior when paired with dipoles as they do when paired with loop coils. Mixing coil types could be complicated in practice, though, since the dipole elements would couple with each other, even if not with the self-decoupled loops. Another observation: it is interesting, and puzzling, that the current distribution in the transmitting dipoles in the figure differs between the two cases of loop-mode and dipole-mode coupling. This indicates that coupling is significantly affecting the behavior of the transmitting partner in the coupled pair, so it is difficult to deconvolve all the relevant effects and simply add up oppositely directed current modes in the self-decoupled loop. At the risk of sounding like a broken record, I would once again make an appeal for some more convincing physical intuition about the mechanism of decoupling.
• Figure 10: It is helpful to show these in vivo results. However, only S parameters and combined images are shown. It would be nice to see individual element images, and to have at least some sense of comparative SAR and/or input power requirements, as a measure of practical performance. Moreover, use of the same coil array for transmit and receive (Methods, lines 392-393) conflates transmit performance with receive performance, making it even harder to judge the overall performance benefits of the self-decoupled design in vivo (see general comment #3 above).

Reviewer #3 (Remarks to the Author):

Although this is a well-written and thorough paper I do not feel that it has the general appeal to a wider audience that Nature Communications strives towards. The approach is novel in itself, but joins a large number of other methods for coil decoupling that have been published in the MR literature. The paper would be much more suitable for a specialized publication such as Magnetic Resonance in Medicine or Journal of Magnetic Resonance.
General points.
1. The authors present results at 7 Tesla, but this remains quite a niche market, and in order to be more relevant to a wider audience it would be much better to at least give some illustrations at either 3 Tesla or 1.5 Tesla.
2. As mentioned by the authors, there are many different schemes for decoupling elements in a receive array, including one by Zhang, Sodickson and Cloos (very recent so understandably not referenced) which also presents a scheme for effective self-decoupling. Although the authors approach in this paper is new, it is not clear that this approach is sufficiently general to “replace” the other ones. Without a strong indication of increased signal-to-noise, for example, compared to other geometries, there is not a very strong rationale for publishing in a high-impact general science journal rather than an MR-specific journal where most of the other designs have been published.
3. Although the concept of self-decoupling is new, there has been quite some previous work on this loophole type of coil (well referenced in this paper), and so again for a high-impact factor journal this does not contain the truly novel nature that one would look for.

Specific points.

1. The mode capacitor has an extremely low value, much less than 1 pF, which normally is a problem with MR coils since parasitic capacitances are at least on the order of this value. Perhaps this explains the greater than 10 dB difference in S21 shown in Figure 10b when the self-decoupled coils are differently loaded? What happens when smaller loops than the current 10 x 10 cm are used, would the mode capacitor value decrease even further? Also, what is the situation at 3 Tesla or 1.5 Tesla which constitutes the vast majority of MRI scanners. Is this approach feasible, and would the required capacitances increase in this case?

2. Figure 2a shows six segmented inductors, Xarm, but the constructed coils in Figure 3a show only one inductor. Does segmentation make any difference except for the absolute values of the inductor(s)? The authors should be consistent in their approach, or at least note whether this makes any difference.

3. Particularly at high field, loops are segmented by a large number of capacitors, and yet the self-decoupled loops (e.g. Figure 3a) have effectively very little or no segmentation. In terms of frequency shifts with different samples, and overall radiation due to long conductor lengths, the authors need to provide more discussion as to why these latter effects are not detrimental to the overall performance of the coil.

4. Figure 10 presents a rather impractical illustration in the sense that one would almost never use a head coil to image the leg (although I understand the point of the robustness with respect to loading). Much more convincing would be to show the SNR of the self-decoupled coil compared to a conventionally decoupled coil, so that the readers could see how much potential advantage they could gain using this new approach.

5. The phantom is placed a long way below the coils, 4.5 cm according to the methods section in line 336, and in practice a distance of more like 2 cm would be used. This suggests that the self-decoupling has a greater advantage over non-decoupled when the coils are lightly loaded. How much does the advantage decrease when they are heavily loaded?

6. (minor) Figure 7 mentions -9.3 dB lower…., whereas the text above the figure mentions -8.3 dB.

Response to Reviewers of "Self-decoupled MRI radiofrequency coils"

We would like to thank the editor and reviewers for their careful and thorough reading of our manuscript and for the constructive suggestions, which helped to improve its quality. Our response follows (reviewer's comments are in italics)

Reviewer #1

R1. 1 *The authors present a method that intrinsically decouples a loop antenna from a neighbouring loop or dipole by equating the current density in the loop and dipole modes of the coil element. Self-decoupled RF coils exhibit excellent decoupling between adjacent and next-nearest-neighbour elements, with robustness to coil loading and the distance between coils.*

Self-decoupled RF coils appear to be a clever means to decouple RF coils without the use of electrical circuits or geometric overlap. This is the first time the difference in current density of loop-mode and dipole-mode coupling has been exploited for this purpose. This technique is certainly original for decoupling RF coils, and is a valuable extension to the loophole introduced by Wiggins et al. This should be of great interest to MRI coil designers, both in academia and industry.

The manuscript is scientifically sound, with no major flaws. The authors address the majority of relevant questions (some further questions are detailed below) regarding a new decoupling scheme-B1+ efficiency, B1+ profile, dependency on load and distance, next-nearest-neighbour coupling, 2D arrays, and local SAR. The methods used to evaluate the decoupling scheme are of high quality and presented in a clear and concise manner. Some additional comments on how to determine Cmode for a large array would be useful to the reader in order to reproduce and implement this decoupling scheme. The abstract and introduction are sufficiently broad and should be accessible to a wide audience. The conclusions are in keeping with the results, although the utility for receive coils may be overstated, as discussed below.

For transmit coils, where geometries are typically simpler than receive coils and often cylindrical, self-decoupled coils should provide a practical means to substantially increase the isolation between coil elements of a loop coil. The ability to produce high isolation between transmit coils, without the necessity of electrical connections or geometric overlap, would be of great use and provide many unique possibilities for transmit coil design. Decoupling transmit coils is still an active area of investigation, and self-decoupling coils presents a potentially large step forward.

When applying the technique to receive coils, the technique is perhaps less useful, for several reasons. First, as geometries become complex, such as for a 32-channel head coil, a single loop will be located at many different angles with its neighbours, thereby causing the designer to compromise in their choice of Cmode; Figure 1 shows a sharp increase in coupling with small changes to Cmode. The authors present a 2x2 array, with still a relatively simple geometry, and yet the decoupling reduces to approximately -15 dB. Although this is still good, it seems as though this would become worse for an increasingly complex geometry where a greater degree of compromise is required when determining Cmode. Furthermore, the decoupling is still not sufficient to make the use of low-input-impedance preamplifiers unnecessary, so I am wary that the use of self-decoupled coils will not change the way receive coils are designed.

A second major drawback for receive coils is the seeming reliance on coil diameters to be relatively large to prevent the need for large, lossy inductors. As mentioned by the authors, meanders could be used, but the space requirements for a high-density receive coil may make this impractical. They also tend to become more lossy unless careful simulation of the meanders is conducted. This is less of an issue for transmit only coils.

A: We appreciate the positive feedback from the reviewers.

1. Regarding self-decoupled coils in complex geometries, such as a 32-channel Rx head coil

We agree that the use of self-decoupling only may not provide sufficient decoupling for dense receive coils with complex geometries. At the same time, the self-decoupled approach can be easily combined with other decoupling method such as element overlapping, shielding and low-impedance preamplifiers.

To address this, we have added the following to the Discussion: “In this report we have emphasized the broad applicability and simplicity of self-decoupled designs by building arrays that used only self-decoupling. Although 2-D and cylindrical arrays are suitable for most applications, close-fitting receive-only 3-D helmet arrays with 32 or more elements may be preferred to capture maximum SNR in brain imaging. For these arrays, the small size, close spacing and varying neighbor geometries may limit the degree of self-decoupling that can be achieved since coils must be decoupled from several neighbors simultaneously. However, self-decoupling can be combined with the most widely used conventional decoupling methods, such as overlapping, low-impedance preamplifiers, shielding, and transformers. For example, in a multi-row array, self-decoupling could be used to decouple elements in the same row and overlapping could be used to decouple elements in different rows.”.

2. Regarding lossy inductors in small self-decoupled coils

We agree that lossy inductors are a possible issue for small self-decoupled coils. Although this problem can be alleviated using dielectric materials or meander lines, these would increase the coils’ complexity. To investigate this further, we made a series of square self-decoupled coils with lengths/widths from 10 cm down to 5 cm in 1 cm steps. We found that the C_{mode} increase approximately linearly as the coil size decreased, and that, for a 5 × 5 cm^2 self-decoupled coil, the C_{mode} was around 0.8 pF and the required inductance X_{arm} was around 80 nH (new Table S2).

“Like conventional loops, the resonance frequency of self-decoupled coils is dominated by the coils’ inductance (self-inductance and X_{arm}) and capacitance. When the coils’ dimension is extremely small compared to the wavelength, setting X_{arm} to a large inductance might be required to maintain tuning. Large lump inductors would have non-negligible resistance, so the performance of extremely small-sized self-decoupled coils might be decreased. This issue could be solved by using dielectric materials with high permittivity to shorten the effective wavelength, or using meander lines to increase the loop’s self-inductance”.

→“Like conventional loops, the resonant frequency of self-decoupled coils is dominated by the coils’ inductance (self-inductance and X_{arm}) and capacitance. The X_{arm} impedance needed to tune a self-decoupled coil’s resonant frequency may be an inductor. In particular, if the C_{mode} capacitor required for self-decoupling remained fixed as the coil size decreases, X_{arm} may have a large inductance which could lead to significant power loss in practice. To give some insight on this matter, we simulated a series of square self-decoupled coils with lengths/widths from 10 cm down to 5 cm in 1 cm steps. We found that the C_{mode} increase approximately linearly as the coil size decreased (Supporting Table S2), and that, for a 5 × 5 cm^2 self-decoupled coil, the C_{mode} was around 0.8 pF and the required total inductance X_{arm} was around 80 nH.”.

<u>Specific comments/suggestions:</u>

R1. 2 *It could be useful to comment on the practical implementation of this procedure. The authors show in Figure 7 that the inclusion of the third coil element changes the optimal Cmode. Given the dependence of Cmode on neighbouring coils, would the entire array be simulated, then Cmode determined from simulation? Or is there a practical method for adjusting Cmode on the bench for a large array of coils?*

A: We have added a specific guide (text in the main body and Supporting Figure S1) on how to tune C_{mode} for two coils based on bench measurements, and we provide guidance on how to extend this practice to a whole array.

For example, we did not simulate the 3-ch array; instead we adjusted the X_{arm} of the middle coil to bring it back the 298 MHz on the bench when the third coil was added.

R1. 3 *For the 2x2 array, the authors state that Cmode was chosen as a compromise for the three coil-combinations causing coupling. If Cmode was not chosen as a compromise, but chosen as the optimal value for each of the three coil combinations, what would the range in Cmode be? Given Figure 1, there seems to be a large increase in coupling with small changes in Cmode. For a head coil that has a spherical-like shape, would this compromise in Cmode render the method unusable? Certainly, for cylindrical coils this is much less of a problem and works quite well.*

A: For the 2 × 2 array, the optimal Channel 1 C_{mode} for same-row decoupling (where the coils partly face each other) was 0.37 pF, and the optimal C_{mode} for different-row decoupling (where the coils do not face each other) was 0.48 pF. A C_{mode} value of ~0.44 achieved acceptable decoupling in both directions. The following table lists Channel 1's optimal C_{mode} in each case and the corresponding decoupling performance. We note that in all three cases C_{mode} was positioned in the corner opposite the feed port, which is suboptimal if only decoupling in one direction. Overall, the range of C_{mode} values is small (0.11 pF).

	C_{mode} (pF)	S21 (dB), coupling between adjacent loops in the same row	S31 (dB), coupling between adjacent loops in different rows
Optimal C_{mode} for same-row decoupling	0.37	-20.7	-13.1
Optimal C_{mode} for different-row decoupling	0.48	-13.4	-19.4
Balanced C_{mode}	~0.44	-17.6	-15.2

For coils on a spherical-like shape, varying neighbor geometries may limit the degree of self-decoupling that can be achieved since coils must be decoupled from several neighbors simultaneously. However, self-decoupling can be combined with the most widely used conventional decoupling methods, such as overlapping, low-impedance preamplifiers, shielding, and transformers. For example, in a multi-row array, self-decoupling could be used to decouple elements in the same row and overlapping could be used to decouple elements in different rows. We have added this comment in the revised manuscript.

R1. 4 *One measurement that is missing is the comparison B_1^+ efficiency (or B_1^- sensitivity) of a single conventional coil to a single self-decoupled coil. It may be sufficient to reference Lakshmanan et al. (Ref. 31) for this; however, it is necessary to show that B1 of s a single coil is not sacrificed (excluding the effects of coupling) by manipulating the current density in this manner.*

A: We agree and have added the measured B_1^+ of a single self-decoupled coil and that of a single self-decoupled coil in Figure 3. There is indeed little sacrifice by using self-decoupled design and two B_1^+ maps are almost the same.

R1. 5 *To compare the B_1^+ efficiency of two coupled conventional coils with two self-decoupling coils is a bit misleading. Some means (either geometric overlap, capacitive or geometric decoupling, etc.) would always be used to decouple conventional coils, which would improve the B_1^+ efficiency.*

A: We have added Supporting Figure S4 which augments Figure 3 by showing the same results using conventional transformer-decoupled (inductively decoupled) coils. Similar to the self-decoupled coil, transformer-decoupled coils also had excellent decoupling performance (around -20 dB) and maintained the original B_1^+ of single conventional coils. However, self-decoupled coils still bear many advantages as described in the text.

R1. 6 *Why is the optimal experimental coupling between a dipole and a self-decoupling loop only -14.8 dB? If the loop-mode and dipole-mode currents cancel, shouldn't the isolation between the two elements be better? Or is this residual coupling due to resistive coupling through the conductive sample?*

A: We indeed believe that the residual coupling is from the resistive coupling, and we have added this comment in the revised manuscript.

R1. 7 *For Figure 6, please explain how S21 is normalized, as this was not clear in the text.*

A: The normalized S21 was defined as $|S21|/(\sqrt{1-|S11|^2})$, which corresponds to S21 in the perfect impedance matching condition. We have added this in the revised manuscript.

R1. 8 *The authors state that self-decoupling coils may become less efficient for smaller coil diameters. Could the authors provide a lower bound for where they feel self-decoupling coils will be useful.*

A: If C_{mode} stayed the same as the coil size decreased, a large inductance would be necessary to maintain resonance, which may decrease coil performance. However, we found that C_{mode} increases as the coil size decreases, so the X_{arm} inductance was acceptable even for a 5 × 5 cm^2 coil, and the ideal B_1 field pattern was maintained. We have added simulation and experimental results of the 5 × 5 cm^2 coil in the revised manuscript. 5 × 5 cm^2 is a reasonable lower bound that meets most applications in human imaging, and state-of-the-art 32-ch and 64-ch head coils have used larger 9.5 cm and 6.5cm diameter coils, respectively (Keil, et al, MRM 70:248–258, 2013).

R1. 9 *In Figure S2, why is there considerably more asymmetry in B1+ as a function of slice number versus the simulation?*

A: This is most likely because we used six X_{arm} (each 5.6 nH) in simulation, but one X_{arm} (around 35 nH) in the constructed coil. We have added this comment in the revised manuscript.

R1. 10 *References to alternative decoupling schemes should be expanded or modified, given this is the primary focus of the manuscript. For decoupling networks, Lee et al. predates the references provided. Pinkerton et al. is the seminal US patent and decoupling method used in commercial scanners (builds on Lee). Methods such as resonant-inductive decoupling (Avdievich et al.) and Cartesian feedback (Hoult et al.) should also be included:*

" Lee RF, Giaquinto RO, Hardy CJ. Coupling and decoupling theory and its application to the MRI phased array. Magn Reson Med 2002;48(1):203-213.

" Pinkerton RG, Barberi EA, Menon RS. Noise properties of a NMR transceiver array coil. J. Magn. Reson. 2004; 171(1):151-156.

" Avdievich NI, Pan JW, Hetherington HP. Resonant inductive decoupling (RID) for transceiver arrays to compensate for both reactive and resistive components of the mutual impedance. NMR Biomed 2013;26(11):1547-1554.

" Hoult DI, Kolansky G, Kripiakevich D, King SB. The NMR multi-transmit phased array: A Cartesian feedback approach. J Magn Reson 2004;171(1):64-70.

A: Thank you for pointing these out. These references have been cited in the revised manuscript.

Reviewer #2

Overall impressions:

This manuscript describes an innovative radiofrequency coil design for magnetic resonance imaging and spectroscopy, addressing the important practical challenge of coupling among elements of a coil array. The self-decoupling design is in principle fairly simple to implement, as a perturbation on traditional coil designs, lending it widespread potential practical value and day-to-day impact. From this point of view, I think that the manuscript would be received with interest and enthusiasm by a broad audience. There are, however, several factors which reduce my enthusiasm for the paper in its current form, including

R2. 1 *The absence of a solid theoretical explanation of the observed decoupling effects, which might help to provide insight into the range of conditions over which favorable effects might be expected.*

A: Thank you for this suggestion. In the revised manuscript, we break down the coupling mathematically into electric (***Ke***) and magnetic (***Km***) components and demonstrated that self-decoupled coils work by canceling these components out. We also quantitatively extracted the two kinds of coupling (Figure 2b), which reveals how magnetic and electric coupling change in different ways as the mode capacitance (C_{mode}) varies. The value of C_{mode} (0.44 pF) predicted from the magnetic/electric analysis is consistent with the real case.

R2. 2 *The authors assert that currents induced by a neighboring coil in the loop-like and dipole-like virtual components of their self-decoupled design are oppositely directed but otherwise equivalent in distribution. This is undoubtedly a convenient property, but it is not at all obvious why this should be the case.*

A: Thank you for pointing this out. We have clarified by changing the statement "These results are consistent with Lenz's law, and are also consistent with prior results on the mutual coupling between two closely-spaced co-linear dipoles (separation distance<1/10 wavelength)" to: "The negative sign of the magnetic coupling coefficients is also consistent with Lenz's law, which dictates that the current induced in the passive coil will generate magnetic flux to cancel out the active magnetic flux through it. As C_{mode} decreases, the two self-decoupled coils increasingly behave as a pair of co-linear folded dipoles that couple electrically, and the positive sign of the electric coupling coefficients is consistent with prior results on the mutual coupling between two closely-spaced co-linear dipoles (separation distance < 1/10 wavelength)[41].".

R2. 3 *In fact, though the simulated current distributions in loop-mode and dipole-mode configurations (e.g. in Fig. 2 and Fig. 4) are indeed oppositely directed, they do not appear to share the same distribution around the coil, and they would therefore NOT cancel one another. The explanation for self-decoupling, then, is not fully satisfying.*

A: Thanks for your comments. In the revised manuscript, we explained the self-decoupling in a different way using the magnetic coupling and electric coupling coefficients.

R2. 4 *One might argue, fairly enough, that the experimental results show value regardless of how convincing the theory may be. However, in the absence of a solid theoretical backdrop, it is difficult to know how generalizable the self-decoupling effect may be in practice. One suggestion might be to separate the self-decoupled coil into its loop-like and dipole-like components, and to perform a separate simulation with each component as a passive second coil paired with the same transmitting coil. I must confess that it is difficult to imagine, based on fundamental symmetries, that the resulting induced current contributions would ever be able to cancel one another, so perhaps there is some fallacy in the superposition argument which remains to be exposed.*

A: 1) In the following figure, we simulated a loop-like coil (C_{mode}= 8pF) and a dipole-like coil (C_{mode}= 8pF) paired with the same transmitting coil (C_{mode}= 0.35 pF). The simulated results are consistent with that in Figure 2.

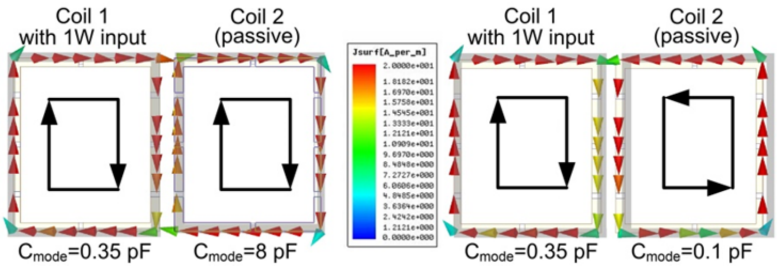

2) In the previous work, we emphasized that the active coil induces two kinds of currents (from dipole-mode coupling and loop-mode coupling) in self-decoupled coils which cancel each other. This is easier for most readers to understand but might lead to confusion. In the revised manuscript, we emphasized that the coupling could be a mix of magnetic coupling (loop-mode) and electric coupling (dipole-mode) which cancel each other. We also extracted the magnetic coupling coefficient ($\boldsymbol{K_m}$) and the electric coefficient (***Ke***) as a function of C_{mode}.

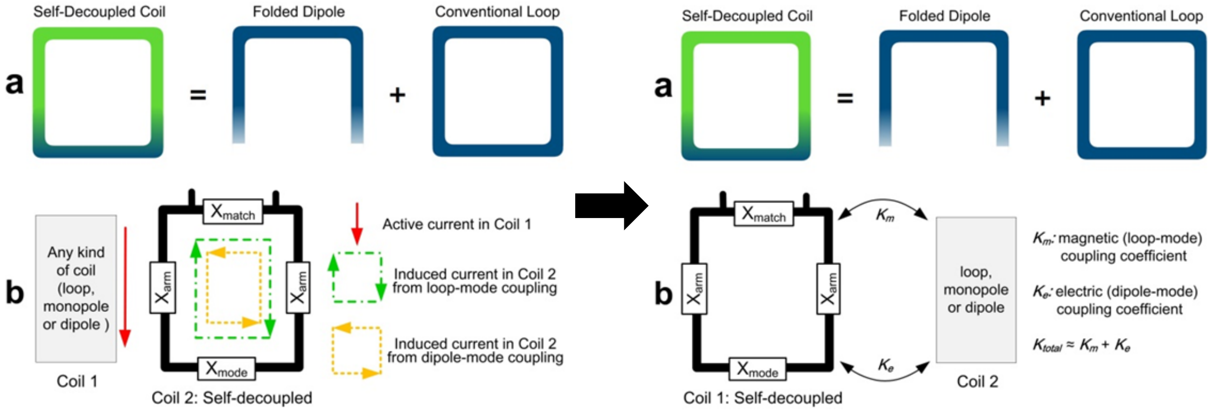

R2. 5 *The fact that a transmitting dipole appears to be decoupled just as well as a transmitting loop from the self-decoupled structure is interesting, and also puzzling. How can the geometry of the transmitting structure not matter?*

A: In the revised manuscript, we explain the self-decoupled coils in a different way using the mixed coupling analysis. Any geometry of transmitting structure can work so long as it can generate the desired electric coupling to cancel the magnetic coupling. We have tried loop (simulation and experiment), dipole (simulation and experiment) and monopole (simulation) coils, and they can all be decoupled from loop coils by self-decoupling.

R2. 6 *Is the properly-tuned self-decoupled structure somehow incapable of carrying a current at all unless it is imposed at the transmit port?*

A: The self-decoupled structure is capable of carrying a current with the absence of the other coil. We think this confusion was caused by the caption of Figure 1b **"**When a current-carrying coil (Coil 1) is placed next to a self-decoupled loop coil (Coil 2), it induces electric currents in the loop via loop-mode coupling (green) and dipole-mode coupling (yellow). The currents circulate in opposite directions, and can be canceled if they are of equal magnitudes, making the coil self-decoupled. ". In the revised manuscript, we explain the self-decoupling in a different way and this caption was modified as: **"**When a self-decoupled loop coil (Coil 1) is placed next to another coil (Coil 2), they couple both magnetically and electrically. If the magnetic and electric coupling coefficients $\boldsymbol{K_m}$ and $\boldsymbol{K_e}$ can be tuned

to have opposite signs and equal magnitudes by adjusting the X_{mode} impedance in Coil 1, the total coupling is zero, and Coil 1 is self-decoupled from Coil 2".

R2. 7 *Simulations like those in Figs. 2 and 4, but with Cmode tuned to the self-decoupled condition, would be interesting to see. Or is the self-decoupled structure in fact a high-impedance structure? One would expect this to result in a low transmit efficiency, however, which does not appear to be the case. Color me curious, but confused.*

A: In the revised manuscript, we added new results (Figure 2e and Figure 4d) with C_{mode} tuned to the self-decoupled condition. Compared to conventional coils, self-decoupled coils indeed have a relatively high-impedance structure due to their small C_{mode} capacitance. The high-impedance structure would induce antenna effects, which might increase radiation loss and reduce transmit efficiency. However, we found that the radiation loss of self-decoupled coils is almost the same as the conventional coils, even at 3T and 1.5T (Supporting Figure S10c). In the revised manuscript, we added the comments: "The radiation losses of the self-decoupled coils were similar to that of conventional coils, even though they behave more like antennas than conventional coils. This means that most of the coils' power was directed into the high-permittivity and conductivity samples, which is consistent with results using straight dipole antennas[51]".

We also noted that X_{arm} with a large inductance is required to maintain coil resonance at low fields (Supporting Figure S10a), which indeed brings notable loss for coils at 1.5T. In the revised manuscript, we added the following comments: "Supporting Figure S10 shows a study of self-decoupled coils at 3 T and 1.5 T, where it was found that the ideal B_1^- fields can be maintained at 3 T for both 10 × 10 cm^2 and 20 × 20 cm^2 coils, but there was an SNR decrease (~20%) for the 10 × 10 cm^2 coil at 1.5 T due to inductor loss. This loss could be avoided by dielectric materials with high permittivity or meander lines, at the cost of increased manufacturing complexity".

2. Certain considerations which might make the self-decoupled design cumbersome in practice, including

R2. 8 *The need to break up the Cmode capacitor to avoid excessive local SAR, resulting in a comparatively large number of closely-spaced lumped elements. This might prove a challenge for flexible coil designs, and might also result in undesired losses for transmit and/or receive efficiency.*

A: The single C_{mode} capacitor could also be replaced as distributed capacitor such as transmission line. If this were done, it would maintain coil flexibility with low local SAR. We have added this comment in the revised manuscript. At the same time, the use of multiple C_{mode} capacitors has little impact on the transmit/receive efficiency since the capacitor loss is negligible compared to the sample loss, even at 3T and 1.5T. In addition, increasing the number of C_{mode} also increases the value of each $C_{mode.}$ Thus the loss of each C_{mode} becomes smaller.

R2. 9 *The need to adjust the position of the Cmode capacitors depending upon the desired decoupling topology. This might be a limitation for the general case of many-element arrays, and it might also influence the transmit and receive sensitivity patterns, which the authors acknowledge will depend upon the orientation of the coils and their ports.*

A: Adjusting of the position of C_{mode} indeed slightly changes the original B_1. At the same time, we found that it may provide a new degree of freedom to improve transmit or receive sensitivity, as shown in the new Supporting Figure S7. When the self-decoupled coil was fed in its vertical conductor, this average B_1 efficiency improvement was 18% for a 10 × 10 cm^2 coil at 7T. We also noted that the self-decoupling method can be easily combined with other methods. If the original B_1 is still preferred in multi-row self-decoupled arrays, combined decoupling can be used to avoid changing the position of C_{mode}, and thus maintain the B_1 distribution of a conventional coil.

R2. 10 *The absence of considerations of receive performance. Although the transmit case is arguably the most*

critical case calling for improved decoupling, receive arrays have a much higher volume of use than transmit arrays, and therefore improvements in receive performance would have a higher overall impact. Reciprocity might lead one to expect receive performance gains that are similar to the gains explored in the transmit setting; however, the devil is often in the details when it comes to RF engineering, and the absence of a clear theoretical backdrop makes it harder than it might otherwise be to rely upon simple intuitions. For example, how would the presence of a low-input-impedance preamplifier affect the decoupling conditions? What about losses in the lumped elements of the self-decoupled design? A detailed exploration of receive effects could legitimately be argued to be beyond the scope of this manuscript, but at least some commentary on expected effects would be welcome.

A: Thanks for your comments. Considering that Review #3 was also concerned with whether self-decoupled coils can be used for small coils, we analyzed the receive performance of a pair of small (5 × 5 cm^2) self-decoupled coils (Supporting Figure S9). We found that the receive sensitivity maps of the self-decoupled coils were nearly the same as those of ideal coils and the coupling between the two self-decoupled coils was -30.1 dB. Since it is still a closed loop structure, we believe the self-decoupled design can be combined with conventional low-impedance preamplifier decoupling. At high fields like 7T, the coil loss is negligible compared to sample loss, but this might be a problem at lower fields. In the revised manuscript, we analyzed the losses in the conductor, lumped elements and samples for self-decoupled and conventional ideal single coils at 3T and 1.5 T (Supporting Figure S10).

On the whole, the manuscript represents a potentially valuable contribution to literature, whose full impact is, however, somewhat difficult to judge without more information.

<u>Additional detailed comments:</u>

R2. 11 *P. 4, lines 55-56: "When receiving RF signals from the body, coupling leads to noise amplification and limits the degree to which a scan can be accelerated using parallel imaging." This is not entirely true. It has been shown that SNR-optimizing reconstructions accounting for noise correlations can undo the effects of moderate degrees of linear inductive coupling. It is only in more extreme cases that the parallel imaging performance, and overall electrical performance, of coupled coils may be degraded. A slightly more nuanced statement might be appropriate.*

A: We agree and have edited this sentence to: "When receiving RF signals from the body, strong coupling can lead to noise amplification and may limit the degree to which image acquisition can be accelerated using parallel imaging."

R2. 12 *Figure 3 and Supplementary Figure 1: It is not entirely clear why the B1+ profile in the self-decoupled case is so close to that in the ideal uncoupled case with uniform currents. The authors make the argument that only two legs of the coil loop are important for B1+ generation, but this is true only in certain coil and slice orientations. A change in current distribution must affect the sensitivity profile in general. Here some simulations of the current distribution in the self-decoupled case – rather than the loop-mode and the dipole-mode cases shown in Figures 2 and 4 – could be helpful.*

A: Thanks for the comments. We have added the current distributions in the self-decoupled case in Figure 2 (loop-loop configuration) and Figure 4 (dipole-loop configuration).We agree that the horizontal conductors indeed contribute to the total B_1 in some specific cases. To investigate this, we simulated a 10 × 10 cm^2 coil and a 15 × 5 cm^2 coil in both light-loading and heavy loading scenarios. Three cases were simulated with unit current source driving: all conductors excited, vertical conductors excited and top horizontal conductor excited. It was found that the horizontal conductor has notable contributions only when the coil is quite wide (15 × 5 cm^2) and placed close the phantom, as shown in the following figure. We have modified this in the revised manuscript: "This can be

understood by considering that the $B_1=(B_x\pm iB_y)/2$ [33] is mainly produced by the current on the vertical conductor segments arms, where the current distribution is relatively uniform”. → “This can be understood by considering that the $B_1^+=(B_x+iB_y)/2$ [42] is mainly produced by the current on the vertical conductor segments arms for this square coil, where the current distribution is relatively uniform.”.

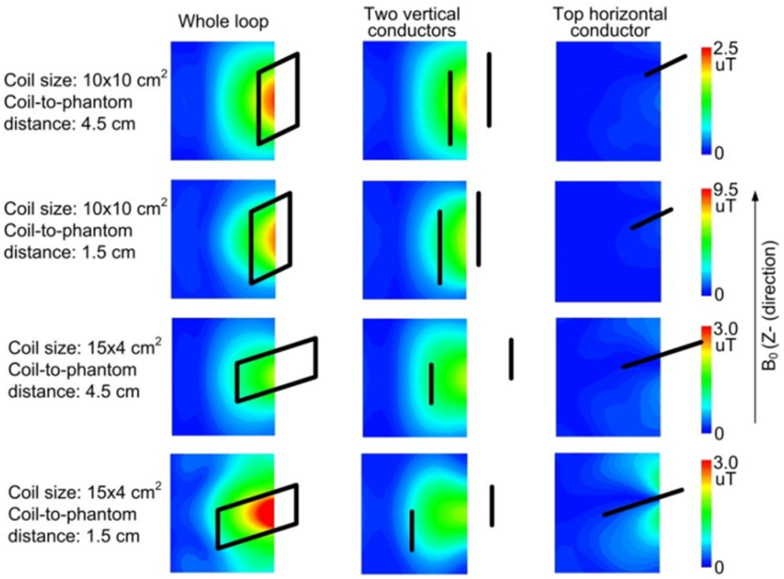

R2. 13 *Figure 4: As mentioned earlier, it is interesting that the self-decoupled coils show similar decoupling behavior when paired with dipoles as they do when paired with loop coils. Mixing coil types could be complicated in practice, though, since the dipole elements would couple with each other, even if not with the self-decoupled loops. Another observation: it is interesting, and puzzling, that the current distribution in the transmitting dipoles in the figure differs between the two cases of loop-mode and dipole-mode coupling. This indicates that coupling is significantly affecting the behavior of the transmitting partner in the coupled pair, so it is difficult to deconvolve all the relevant effects and simply add up oppositely directed current modes in the self-decoupled loop. At the risk of sounding like a broken record, I would once again make an appeal for some more convincing physical intuition about the mechanism of decoupling.*

A: As discussed above, in the revised manuscript we emphasized that the coupling could be a mix of magnetic coupling (loop-mode) and electric coupling (dipole-mode) which cancel each other.

We agree that coupling between adjacent dipoles might be a concern in mixed arrays. This topic is beyond the scope of this manuscript, but we added the following comments: "Another concern for mixed arrays is coupling between adjacent dipole/monopole elements. However, several methods have been described to reduce coupling between closely spaced dipoles/monopoles, such as passive antennas[48,49] and metamaterials[50]. This report focused on the characterization and validation of self-decoupling itself, and further work is needed to investigate potential combinations with these methods.”

R2. 14 *Figure 10: It is helpful to show these in vivo results. However, only S parameters and combined images are shown. It would be nice to see individual element images, and to have at least some sense of comparative SAR and/or input power requirements, as a measure of practical performance. Moreover, use of the same coil array for transmit and receive (Methods, lines 392-393) conflates transmit performance with receive performance, making it even harder to judge the overall performance benefits of the self-decoupled design in vivo (see general comment #3 above).*

A:	Thanks for your comments. We have added B_1^+ maps and MR images from individual coils in the revised manuscript. We also reported the required input power compared to a widely-used commercial transmit array (Nova). The vendor did not provide the SAR results so it is hard to make this comparison. However, we have demonstrated that the maximum local SAR of a self-decoupled coil is similar to that of an ideal single conventional coil. We agreed that both transmit and receive performance should be reported. In the revised manuscript, we added the receive sensitivity maps of a 2-channel self-decoupled array (Supporting Figure S9) and its comparison with ideal single conventional coils. The in vivo result can be seen as an extension of Figure 2, as further evidence for excellent decoupling performance.

<u>Reviewer #3:</u>

R3. 1 *Although this is a well-written and thorough paper I do not feel that it has the general appeal to a wider audience that Nature Communications strives towards. The approach is novel in itself, but joins a large number of other methods for coil decoupling that have been published in the MR literature. The paper would be much more suitable for a specialized publication such as Magnetic Resonance in Medicine or Journal of Magnetic Resonance.*

A:	There is certainly a large body of work on coil decoupling in the MR literature. At the same time, we believe this paper is well-suited for Nature Communications because it describes a method that is very simple to understand and implement, yet can have a widespread impact on current practice.

General points.

R3. 2 *The authors present results at 7 Tesla, but this remains quite a niche market, and in order to be more relevant to a wider audience it would be much better to at least give some illustrations at either 3 Tesla or 1.5 Tesla.*

A:	We agree and have added simulation results at 3T and 1.5T in the revised manuscript (Supporting Figure S10). The required C_{mode} increased at low field, and the simulated receive sensitivity of at 3 T is almost same to the ideal single RF coil (<2%). At 1.5T, the large inductance of X_{arm} induced loss which led to a receive sensitivity decrease.

R3. 3 *As mentioned by the authors, there are many different schemes for decoupling elements in a receive array, including one by Zhang, Sodickson and Cloos (very recent so understandably not referenced) which also presents a scheme for effective self-decoupling. Although the authors approach in this paper is new, it is not clear that this approach is sufficiently general to “replace” the other ones. Without a strong indication of increased signal-to-noise, for example, compared to other geometries, there is not a very strong rationale for publishing in a high-impact general science journal rather than an MR-specific journal where most of the other designs have been published.*

A:	Thanks for this information. The Zhang manuscript is certainly a relevant citation and we will be happy to reference it once it is published in peer-reviewed form. It also represents a major development in the field, but it requires new coil construction approaches, while self-decoupling can be immediately integrated with existing coil designs. Thus, we believe both approaches have their distinct merits.

R3. 4 *Although the concept of self-decoupling is new, there has been quite some previous work on this loophole type of coil (well referenced in this paper), and so again for a high-impact factor journal this does not contain the truly novel nature that one would look for.*

A: The self-decoupled coil focuses on the decoupling performance of RF array coils (both transmit and receive). The novelty is that the RF coil can be self-decoupled by simply changing the impedance distribution to make the magnetic coupling and electric coupling cancel each other, which makes it uniquely able to exhibit robust decoupling performance versus coil distance and reduce coupling between non-adjacent elements and between loops and other element types in mixed arrays.

To clarify the distinction between self-decoupled coils and "loopole" coils, we added following to the Discussion: "Self-decoupled loop coils are built with unequal capacitance and inductance distributions to intentionally generate electric coupling that cancels magnetic coupling. They are distinct from "loopole" coils[38], in which vertical conductors are built with unequal capacitance distributions to generate unequal current distributions that approximate transmit- or receive-optimal current patterns at 7T. However, as shown in Supporting Figure S7, if a self-decoupled coil is rotated by 90 degrees or fed at its corner, the currents on its two vertical conductors become different so its B_1 field becomes more similar to that of a "loopole" coil [38,44], which may be helpful to improve transmit or receive efficiency. It should be noted that this may increase coupling with dipole or monopole antennas in the same array. More broadly, self-decoupled coils should enable greater flexibility in RF array design, since they alleviate constraints on element overlap and spacing. As an example, Supporting Figure S8 shows how decoupling can be made independent of overlapping area using the self-decoupled approach, which provides a new degree of freedom for RF array design to optimize parallel imaging performance and imaging coverage. "

Specific points.

R3. 5 *The mode capacitor has an extremely low value, much less than 1 pF, which normally is a problem with MR coils since parasitic capacitances are at least on the order of this value. Perhaps this explains the greater than 10 dB difference in S21 shown in Figure 10b when the self-decoupled coils are differently loaded? What happens when smaller loops than the current 10 x 10 cm are used, would the mode capacitor value decrease even further? Also, what is the situation at 3 Tesla or 1.5 Tesla which constitutes the vast majority of MRI scanners. Is this approach feasible, and would the required capacitances increase in this case?*

A: Thanks for your comments. The parasitic capacitance could be a reason for the changes in S21 of the self-decoupled coils. However, this does not only happen for the self-decoupled coils. As shown in Figure 6, the S21 of conventional overlapped coils changes around 10 dB when are differently loaded as well.

We also added a study on coil size at 7T, which showed that the mode capacitance (C_{mode}) increased approximately linearly as the loop size decreased. For example, the required C_{mode} increased from 0.44 pF to 0.8 pF when the loop's size decreased from 10 × 10 cm^2 to 5 × 5 cm^2. In the revised manuscript, we added this and experimental results of a pair of small-size self-decoupled coils (5 × 5 cm^2). The C_{mode} also increases as the static magnetic field decreases. For example, the C_{mode} of a 10 × 10 cm^2 self-decoupled coil increases from 0.44 pF to 1.0 pF when the static magnetic field deceases from 7T (Larmor frequency 298 MHz) to 3T (Larmor frequency 128 MHz). The increased C_{mode} reduces the increase in necessary X_{arm} inductance to maintain tuning. We have added simulation results showing this at 3T and 1.5T (Supporting Figure S10).

R3. 6 *Figure 2a shows six segmented inductors, Xarm, but the constructed coils in Figure 3a show only one inductor. Does segmentation make any difference except for the absolute values of the inductor(s)? The authors should be consistent in their approach, or at least note whether this makes any difference.*

A: We used six inductors (X_{arm}) in our simulations to maintain generality. For the 10 × 10 cm^2 coil, we found that the value of each X_{arm} was only 5.6 nH, so one inductor with a value of around 35 nH was used when building it. Also, to avoid the confusion with the transformer decoupling method which uses a pair of close placed windings, the inductors in the two coils were placed far away from each other. We have added these comments in the revised manuscript.

R3. 7 *Particularly at high field, loops are segmented by a large number of capacitors, and yet the self-decoupled loops (e.g. Figure 3a) have effectively very little or no segmentation. In terms of frequency shifts with different samples, and overall radiation due to long conductor lengths, the authors need to provide more discussion as to why these latter effects are not detrimental to the overall performance of the coil.*

A: There are two reasons that loops are preferred to be segmented by a large number of capacitors: the first is to avoid the antenna effect which lead to high coil/radiation loss, and the second is to avoid large frequency shifts with different samples. As for the antenna effect, the self-decoupled coil might have higher radiation loss in free space since it is a combination of a conventional loop and a folded dipole antenna. However, it has only a small radiation power loss when loaded by a high-permittivity phantom or tissues (Supporting Figure S10c). As for the frequency shifts, we did find that self-decoupled coils have a larger frequency shift compared to conventional loop coil, especially when the coil was placed close to phantom. However, we also found its matching performance versus loading is similar to that of a conventional coil. We have added comments in the Results and Discussion on these points.

R3. 8 *Figure 10 presents a rather impractical illustration in the sense that one would almost never use a head coil to image the leg (although I understand the point of the robustness with respect to loading). Much more convincing would be to show the SNR of the self-decoupled coil compared to a conventionally decoupled coil, so that the readers could see how much potential advantage they could gain using this new approach.*

A: We have removed the in vivo leg images, and added results on the receive performance of a self-decoupled coil in comparison with ideal single conventional coils. The self-decoupled could achieve almost the same B_1^+ and B_1^- performance as ideal single coils. Although the two-channel transformer-decoupled coil also achieved similar performance, the self-decoupled coil still has the unique merits that it exhibits robust decoupling performance versus coil separation, it does not require physical connections, and it can be used mixed dipole/monopole+loop arrays as well as loop-only arrays.

R3.9 *The phantom is placed a long way below the coils, 4.5 cm according to the methods section in line 336, and in practice a distance of more like 2 cm would be used. This suggests that the self-decoupling has a greater advantage over non-decoupled when the coils are lightly loaded. How much does the advantage decrease when they are heavily loaded?*

A: A coil-to-phantom distance of 4.5 cm was used since it corresponds to Tx-only coil designs and is much more challenging in practice. We agree that the advantage is not that much in heavy loading case since the coupling becomes lower even for non-decoupled coils. In the revised manuscript (Supporting Figure S9, 5 × 5 cm^2 coils), we show results of a heavy loading case (coil-to-phantom distance 1 cm). Compared to non-decoupled coils, the self-decoupled coils still had a decoupling improvement of -30.1 dB vs. -6.8 dB and a B_1^+ efficiency improvement of 37% and 21%.

R3. 10 *(minor) Figure 7 mentions -9.3 dB lower…., whereas the text above the figure mentions -8.3 dB.*

A: Thanks for pointing out this typo. It has been corrected.

Reviewers' comments:

Reviewer #1 (Remarks to the Author):

My critiques have been addressed satisfactorily.

Reviewer #2 (Remarks to the Author):

• My principal concern with the original version of the manuscript was the absence of a convincing theoretical framework for understanding the proposed decoupling effects. The authors have provided just such a framework in the current revision. They argue that the self-decoupling coil structures arrange a cancellation between magnetic coupling effects (Km) and electric coupling effects (Ke). Though this may have been implicit in their earlier explanations, it is now much clearer and more convincing. Thank you!
• In light of this, and their other thoughtful responses to critiques, I now support publication.
• Here are just a few additional minor suggestions for the authors to take into consideration
o Figure 2: 'Magnetic' and 'electric' labels appear to be switched in panel 2d.
o P. 13, lines 215-218: "For the self-decoupled coils, however, good matching (<-22 dB) and decoupling performance (<-20 dB) were maintained across coil separations. This
can be understood by considering that both the electric coupling and magnetic coupling vary similarly as the coil-to-coil distance varies, so they always cancel each other out." This is a helpful explanation of why the self-decoupling condition can be maintained over varying coil separations. It would be great to see a plot of Ke and Km vs separation, to support this statement. If such a plot is difficult to produce, I would by no means insist on it. However, if it is not too much of a burden, it would really drive the point home nicely. This is particularly true because there is a famous apparent counterexample to combat: in Fig. 9 of Roemer's classic 1990 paper, Ke and Km change very differently with coil separation, and they never exactly cancel. However, Roemer's definition of Ke is, essentially, resistive coupling through the sample (or, equivalently, noise correlation), which is almost certainly different than the definition of Ke used here. A brief comment to this effect would be welcome. Once again, it is not necessary – the results on decoupling independent of separation do speak for themselves, and the authors need not do all the heavy lifting for the readers here – but it would certainly be interesting and helpful.
o The article outlining an alternative decoupling approach based on intrinsically high impedance structures, mentioned by Referee 3 in comment R3.3, has recently been published: https://www.nature.com/articles/s41551-018-0233-y. Personally, I don't think much in the way of detailed commentary or comparison is called for, given the different mechanisms in play, but it does at least help to highlight the importance of and interest in the problem of coupling.

Reviewer #3 (Remarks to the Author):

The authors have provided an exhaustive reply to all of the reviewer comments and questions and have substantially strengthened the paper. With respect to my specific questions I have only one general and two specific remarks/questions.

General. It seems as if the approach is ideally suited for a one-dimensional array, and therefore as pointed out by one of the other reviewers to a transmit array. The authors state that one can form a two-dimensional array by combining this new approach with overlapping or other decoupling method, but this largely removes the relevance and advantage of this new method. I think it is important to be more definitive in terms of being clear whether the new approach absolutely cannot be used in large multi-dimensional arrays or that it just becomes very difficult to

implement it.

R3.6. It is still a little unclear to me why the authors simulate with 6 inductors and construct with one (i don't understand the comment about maintaining generality). I am sure it does not make much difference to the results, but i would like to see a simulation corresponding to the physical coil. Certainly the authors can then comment that the inductor can be split into multiple elements and the results do not change much.

R3.7. The authors have indeed confirmed that there is a larger frequency shift from the self-decoupled coils than for more conventional approaches, and typically this means that matching and therefore sensitivity is very distance-dependent, in addition to the absolute SNR being somewhat lower due to higher electric field coupling to the sample. Can the authors give some idea about how big these frequency shifts are? (line 226)

Response to Reviewers of "Self-decoupled MRI radiofrequency coils. R1"

We would like to thank the editor and reviewers for their careful and thorough reading of our manuscript and for the constructive suggestions, which helped us further improve its quality. Our response follows (reviewer's comments are in italics).

***Reviewer #1** (Remarks to the Author):*

My critiques have been addressed satisfactorily.

Reviewer #2 (Remarks to the Author):

My principal concern with the original version of the manuscript was the absence of a convincing theoretical framework for understanding the proposed decoupling effects. The authors have provided just such a framework in the current revision. They argue that the self-decoupling coil structures arrange a cancellation between magnetic coupling effects (Km) and electric coupling effects (Ke). Though this may have been implicit in their earlier explanations, it is now much clearer and more convincing. Thank you! In light of this, and their other thoughtful responses to critiques, I now support publication. Here are just a few additional minor suggestions for the authors to take into consideration.

A: Thank you for this comment. We are happy that we were able to address this question and significantly strengthen the manuscript.

2.1 Figure 2: 'Magnetic' and 'electric' labels appear to be switched in panel 2d.

A: We have corrected this typo.

2.2 P. 13, lines 215-218: "For the self-decoupled coils, however, good matching (<-22 dB) and decoupling performance (<-20 dB) were maintained across coil separations. This can be understood by considering that both the electric coupling and magnetic coupling vary similarly as the coil-to-coil distance varies, so they always cancel each other out." This is a helpful explanation of why the self-decoupling condition can be maintained over varying coil separations. It would be great to see a plot of Ke and Km vs separation, to support this statement. If such a plot is difficult to produce, I would by no means insist on it. However, if it is not too much of a burden, it would really drive the point home nicely. This is particularly true because there is a famous apparent counterexample to combat: in Fig. 9 of Roemer's classic 1990 paper, Ke and Km change very differently with coil separation, and they never exactly cancel. However, Roemer's definition of Ke is, essentially, resistive coupling through the sample (or, equivalently, noise correlation), which is almost certainly different than the definition of Ke used here. A brief comment to this effect would be welcome. Once again, it is not necessary – the results on decoupling independent of separation do speak for themselves, and the authors need not do all the heavy lifting for the readers here – but it would certainly be interesting and helpful.

A: Thank you for this comment. When the coils are self-decoupled, i.e., K_m and K_e cancel each other, f_m (the transmission zero frequency), f_{even} (the even mode resonance frequency) and f_{odd} (the odd mode resonance frequency) are almost the same. Under this condition, it can be seen from Equations A1 and A2 (repeated below) that the denominators in the K_m and K_e equations are close to zero, which makes it difficult to accurately extract K_m and K_e from simulation:

$$|K_m| = \frac{1}{2}\left(\frac{f_{odd}^2 - f_0^2}{f_{odd}^2 - f_m^2} + \frac{f_{even}^2 - f_0^2}{f_m^2 - f_{even}^2}\right) \qquad \text{(A1)}$$

$$|K_e| = \frac{f_m^2}{2f_0^2}\left(\frac{f_0^2 - f_{odd}^2}{f_m^2 - f_{odd}^2} + \frac{f_0^2 - f_{even}^2}{f_{even}^2 - f_m^2}\right) \qquad \text{(A2)}$$

As the reviewer correctly points out, our K_m and K_e vary differently compared to Roemer's paper, partly because a folded dipole mode is induced and partly because K_e as defined here is slightly different from Roemer's K_e definition. We have added some comments in the revised manuscript to explain this: "Note that the K_e definition used here represents electric coupling via the free space, and unlike the definition used in Roemer et al, it is primarily a reactive coupling and does not include resistive coupling through the conductive sample". Please note that in Figure 2b, while we were able to calculate K_e and K_m across a range of C_{mode} values, due to this same numerical issue we were not able to calculate it precisely at the self-decoupled point.

2.3 The article outlining an alternative decoupling approach based on intrinsically high impedance structures, mentioned by Referee 3 in comment R3.3, has recently been published: https://www.nature.com/articles/s41551-018-0233-y. Personally, I don't think much in the way of detailed commentary or comparison is called for, given the different mechanisms in play, but it does at least help to highlight the importance of and interest in the problem of coupling.

We agree, and have cited this paper in the revised manuscript and noted its mechanism.

<u>Reviewer #3:</u>

The authors have provided an exhaustive reply to all of the reviewer comments and questions and have substantially strengthened the paper. With respect to my specific questions I have only one general and two specific remarks/questions.

General. It seems as if the approach is ideally suited for a one-dimensional array, and therefore as pointed out by one of the other reviewers to a transmit array. The authors state that one can form a two-dimensional array by combining this new approach with overlapping or other decoupling method, but this largely removes the relevance and advantage of this new method. I think it is important to be more definitive in terms of being clear whether the new approach absolutely cannot be used in large multi-dimensional arrays or that it just becomes very difficult to implement it.

A: We agree with the reviewer that readers may be confused by this question. While we did demonstrate that we could build a decoupled two-dimensional (2-D) array using self-decoupling only (Figure 8), we also stated that one might need to combine self-decoupling with other methods to build a three-dimensional (3-D) helmet coil array. We expect that with further optimization self-decoupling may be able to solve the complex coupling issues in a 3-D coil array, but this is beyond the scope of this manuscript. We have added the following comment in the revised manuscript: "Further optimization of self-decoupled coils may be needed to solve these complex coupling issues".

At the same time, we stated in the manuscript that "For example, in a multi-row array, self-decoupling could be used to decouple elements in the same row and overlapping could be used to decouple elements in different rows." This may cause readers to conclude that the method is only useful in one dimension, so we have modified it to: "Alternatively, the self-decoupling design described here can be readily paired with conventional decoupling methods such as overlapping, low-impedance preamplifiers, shielding, and transformers, to address coupling in arrays with complex geometries. In particular, for 3D receive arrays self-decoupling can be combined with preamplifier decoupling to achieve high isolation between all neighbors."

Previously, Referee #1 noted that self-decoupling might not be well-suited to decoupling arrays of small coils (such as are found in 3D helment arrays) due to possibly large X_{arm} inductors with non-negligible resistance. However, in our previous revision we demonstrated that the X_{arm} inductors remain small in small-sized self-decoupled coils

because the C_{mode} values increase as coil size decreases. We also demonstrated that the receive sensitivities of small self-decoupled coils are nearly the same as ideal conventional single coils (Supplementary Figure S9).

R3.6. It is still a little unclear to me why the authors simulate with 6 inductors and construct with one (I don't understand the comment about maintaining generality). I am sure it does not make much difference to the results, but I would like to see a simulation corresponding to the physical coil. Certainly the authors can then comment that the inductor can be split into multiple elements and the results do not change much.

A: We apologize for the confusion on this point. To clarify, Figure 2 shows simulations of a series of coils with different values of C_{mode}. The corresponding total X_{arm} for different C_{mode}s varied significantly. In cases where C_{mode} was close to zero, the X_{arm} was as high as 150 nH. For such large inductances, it would not be practical to use one inductor since it might cause a locally large current. For this reason, six inductors in series were used in the simulations to ensure that the current was relatively uniform. For the constructed two-element self-decoupled coil array, only one inductor was used in each coil because the total X_{arm} was only ~35 nH. To verify that there would be no significant difference between these coils and coils with six inductors, we simulated them with one and six inductors. Their S12's were both ~-20 dB and their B_1^+ fields (central transverse slice shown below) were almost identical.

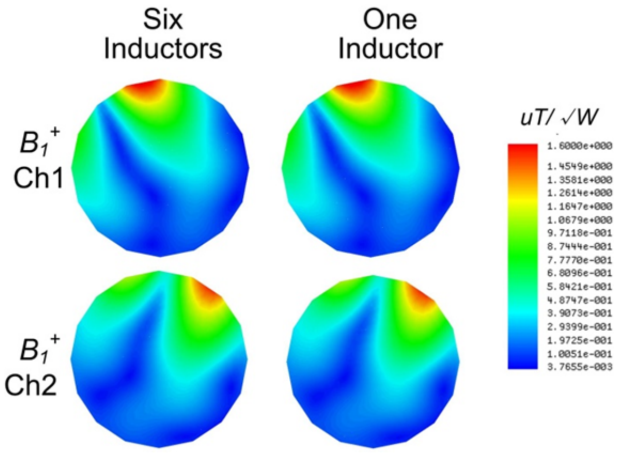

R3.7. The authors have indeed confirmed that there is a larger frequency shift from the self-decoupled coils than for more conventional approaches, and typically this means that matching and therefore sensitivity is very distance-dependent, in addition to the absolute SNR being somewhat lower due to higher electric field coupling to the sample. Can the authors give some idea about how big these frequency shifts are? (line 226)

A: We agree that the frequency shift results indicate that matching is distance-dependent. However, we found that both self-decoupled and conventional coils' matching were distance-dependent, and that although the self-decoupled coil had a larger frequency shift with varying distance, the two coil types had similar overall matching performance across distances. We have revised the manuscript as follows: "Although they experienced larger frequency shifts with varied loading,…"→ "Although they experienced larger frequency shifts with varied loading (7.3 MHz vs. 3.6 MHz when the coil-to-phantom distance changed from 4.5 cm to 1.5 cm),…".

REVIEWERS' COMMENTS:

Reviewer #3 (Remarks to the Author):

The authors have answered my remaining questions completely and so if the other two referees both suggest publication that would also be my recommendation.
